# Boost piezocatalytic activity of BaSO_4_ by coupling it with BaTiO_3_, Cu:BaTiO_3_, Fe:BaTiO_3_, S:BaTiO_3_ and modify them by sucrose for water purification

**DOI:** 10.1038/s41598-022-24992-y

**Published:** 2022-12-01

**Authors:** Omid Amiri, Gashaw L. Abdulla, Chnar M. Burhan, Hawnaz H. Hussein, Amir Mahyar Azhdarpour, Mohsen Saadat, Mohammad Joshaghani, Peshawa H. Mahmood

**Affiliations:** 1 Faculty of Chemistry, Razi University, Kermanshah, 67149 Iran; 2 Chemistry Department, College of Science, University of Raparin, Rania, Kurdistan Region Iraq; 3 Applied Geological Research Center of Iran, Karaj, Iran; 4 Department of Physics, University of Sistan and Baluchestan, Zahedan, Iran

**Keywords:** Environmental sciences, Chemistry, Materials science

## Abstract

The purpose of this study is to improve the efficiency of decontamination using BaSO_4_ as a piezocatalyst. Three techniques are employed in this study to enhance the piezocatalytic activity of BaSO_4_. The first method involves coupling BaSO_4_ with BaTiO_3_. The acid red 151 and acid blue 113 decontamination rates improved from 56.7% and 60.9% to 61.3% and 64.4%, respectively, as a result of this strategy. Additionally, the composite of BaSO_4_ and BaTiO_3_ was doped with copper, iron, sulfur, and nitrogen. By doping BaTiO_3_, acid red 151 and acid blue 113 achieved 86.7% and 89.2% efficiency, respectively. Finally, the nanostructures were modified with sucrose. These strategies improved degradation efficiency for acid red 151 and acid blue 113 to 92.9% and 93.3%, respectively. The reusability results showed that the piezo-catalytic activity of the m-S–BaSO_4_–BaTiO_3_ catalyst did not show a significant loss after five recycles for the degradation of AB113.

## Introduction

Water containing organic pollutants such as phenolic compounds, dyes, and antibiotics is almost non-biodegradable. Such toxic pollutants create chronic toxicity and sometimes can be carcinogenic. These unwelcome properties cause an enormous challenge to the environmental amendment^1–5^. Therefore, it is an urgent demand to treat and neutralize wastewater and constrain the deterioration of water quality to reduce the risks stood to creatures and bio networks^6^. However, conservative treatment approaches such as photocatalysis, photolysis, Fenton Process, and ozonation have some disadvantages such as proper pH/temperature and slow reaction rate^7–9^. So far, advanced oxidation technologies (AOT) have been successfully applied to remove toxic materials^10–12^. Organic contaminants have been decontaminated and decomposed using semiconductor materials in AOT. During this process, strong oxidizing radicals are generated when visible to ultraviolet wavelength light is illuminated. The free radicals produced by these processes react with toxic pollutants^13,14^. In typically advanced oxidation catalysis, the semiconductor should have a vigorous capacity to generate separated electron–hole pairs on the surface by irradiation of photons with energy more than its bandgap. However, the rapid charge carrier’s recombination, a low photon-to-current yield of semiconductor photocatalysts, and low percentages of UV light in the sunlight lead to a low level of photocatalytic efficiency for practical application. Therefore, researchers look for alternative clean and renewable energy to treat wastewater. Piezo-catalytic degradation is a viable alternative to photocatalytic degradation in environmental remediation.

The piezoelectric material can produce electrons and holes by harvesting energy from mechanical vibrations in the surrounding environment. Using these electrons and holes, oxidative free radicals can be produced to decontaminate water^15–18^. From an environmental perspective, lead-free piezoelectric materials are of particular interest^19,20^. Due to its non-toxic structure and abundance^21,22^, BaSO_4_ might be an interesting candidate. However, BaSO_4_ suffers from low piezoelectricity. By coupling it with doped and non-doped BaTiO_3_ and preparing related composites with sucrose, we attempted to improve its piezocatalytic activity. By using dopants, researchers have been able to enhance piezoelectric coefficients. As an example, Shruti B. Seshadri et al. Al. reported a huge enhancement in piezoelectric coefficients and piezoelectricity of lead zirconate titanate by doping 2% of Sm^23^. Another report published by H. M. A. Hamid, and Z. Çelik-Butler demonstrated that the piezoelectricity of ZnO could improve by doping with Li-ion^24^. Doping BaSO_4_–BaTiO_3_ with a dopant can potentially improve its performance because of the following reasons. First, the dopant could act as a shallow-level acceptor in BaSO_4_–BaTiO_3_ and can significantly reduce the piezoelectric potential screening effect^25–29^. Second, depending on the radius of the dopant, it could create an increased strain while replacing the Ba with the BaSO_4_–BaTiO_3_ lattice, thus leading to an increase in the piezoelectric coefficient^30–32^. Next, dopants could increase electrical resistivity and reduce charge leakage^33^.

This research aims to enhance the piezocatalytic activity of BaSO_4_ by coupling it with BaTiO_3_ (doped and non-doped) and sucrose. One of the most common natural piezoelectric materials is sucrose. BaSO_4_, BaSO_4_–BaTiO_3_, doped BaSO_4_–BaTiO_3_, and BaSO_4_–BaTiO_3_-Sucrose composites were used to treat water containing various contaminants. As a mechanical source, ultrasonic vibrations were used to stimulate the piezo material. The results indicate that coupling BaTiO_3_ and sucrose has a dramatic effect on its piezocatalytic activity. In terms of solving environmental problems, piezocatalysts appear to be a viable alternative to AOP technology. Additionally, the effects of pulse and power of ultrasonics on the decontamination efficiency of organic pollutants were investigated.

## Experimental

### Material

Synthesis of piezocatalyst: For bare BaSO_4_–BaTiO_3_ without dopant, 1.19 g BaSO_4_ was dispersed in 10 mL distilled water. Then 11 mL of an ethanol-based solution of tetraethyl orthotitanate was added to the above solution and stirred for 10 min. Then 2 mL of NaOH 0.5 M was added under stirring. Then the solution was transferred to an autoclave and was heated at 160 °C for 8 h. Finally, the obtained precipitate was washed twice with ethanol and water and calcinated at 750 °C for 2 h.

For doped BaSO_4_–BaTiO_3_, 1.19 g BaSO_4_ was dissolved in 10 mL dispersed water. Afterward, 0. 12 mmol of a dopant was added to the initial solution. Thioacetamide, copper sulfate, iron sulfate, or ammonia was added as a dopant. 11 mL of the ethanol-based solution of tetraethyl orthotitanate (10% V:V) was added to the above solution and stirred for 10 min. Then 2 mL of NaOH 0.5 M was added under stirring. Then the solution was transferred to an autoclave and was heated at 160 °C for 8 h. Finally, the obtained precipitate was washed twice with ethanol and water and calcinated at 750 °C for 2 h.

#### Preparing doped BaSO_4_–BaTiO_3_-sucrose

1 g of doped BaSO_4_–BaTiO_3_ was added to the 20 mL DI water. Then 20 mL of an aqueous solution of sucrose (0.2 M) was added to the above solution and stirred for 12 h. The above solution was centrifuged for 10 min to remove an excess of sucrose.

#### Piezocatalytic decontamination test

The piezoelectric catalytic performance of the doped and modified BaSO_4_–BaTiO_3_ was evaluated by their ability to degrade acid red 151 (AR151) as an organic pollutant. In each piezocatalytic experiment, 50 mg of a BaTiO_3_-based catalyst was dispersed in a 100 mL beaker containing 50 mL AR151 solution (5 ppm).

Before the degradation process, the mixture was magnetically stirred for 30 min in the dark until adsorption–desorption equilibrium was attained. Then the UV–Vis absorption spectra of the samples were recorded just before turning on ultrasound. The experiment was performed in the darkness to eliminate the interference of light. The piezocatalytic performance of the prepared samples was tested by the degradation of AR151 and acid blue 113 (AB113) under ultrasonic vibration. Here, ultrasonic prob with power of 100 W and ultrasound frequency of 20 kHz was used for 60 min as mechanical source. The mixture was centrifuged and the concentration of AR151 and AB113 was measured from their UV–Vis absorbances. Besides, we studied the effect of vibration pulse and power.

## Results and discussion

Here we improved the efficiency of the piezocatalytic activity of BaSO_4_–BaTiO_3_ as a new class of catalyst for the decontamination of water. This is a promising way to use mechanical waste energy to treat wastewater. Here we doped BaSO_4_–BaTiO_3_ by Cu, Fe, S, and N and coupled them with sucrose as a natural piezomaterial that was labeled as Cu–BaSO_4_–BaTiO_3_, Fe–BaSO_4_–BaTiO_3_, S–BaSO_4_–BaTiO_3_, N–BaSO_4_–BaTiO_3_, Cu–BaSO_4_–BaTiO_3_-sucrose, Fe–BaSO_4_–BaTiO_3_-sucrose, S–BaSO_4_–BaTiO_3_-sucrose, and N–BaSO_4_–BaTiO_3_-sucrose, respectively.

**Figure 1:**
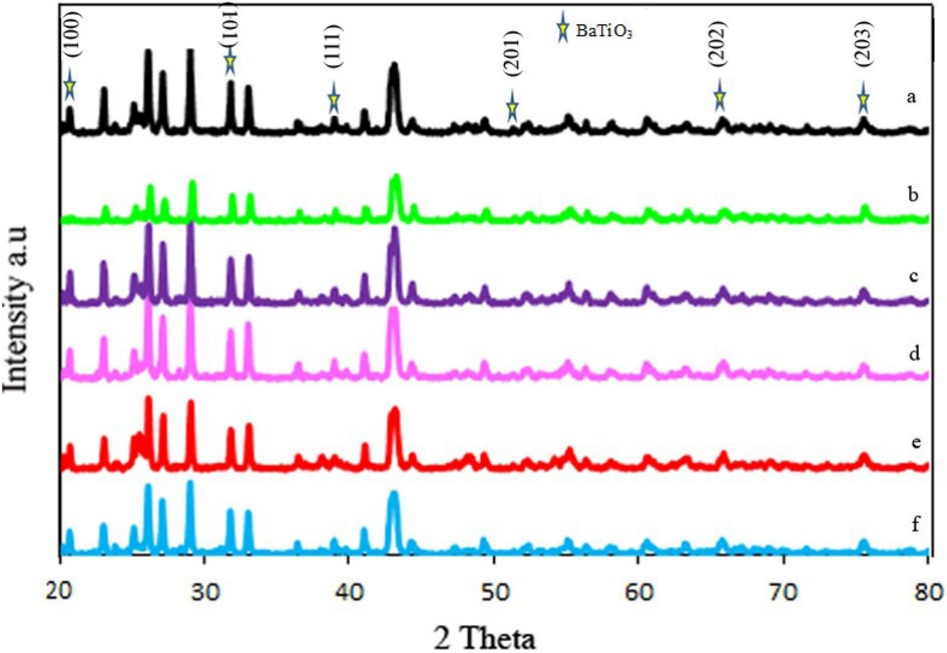
XRD pattern of (**a**) BaSO_4_–BaTiO_3_, (**b**) Cu–BaSO_4_–BaTiO_3_, (**c**) Fe–BaSO_4_–BaTiO_3_, (**d**) S– BaSO_4_–BaTiO_3_, (**e**) N–BaSO_4_–BaTiO_3_, and (**f**) m-S–BaSO_4_–BaTiO_3_.

The XRD patterns of BaSO_4_–BaTiO_3_, Cu–BaSO_4_–BaTiO_3_, Fe–BaSO_4_–BaTiO_3_, S–BaSO_4_–BaTiO_3_, N–BaSO_4_–BaTiO_3_, and m-S–BaSO_4_–BaTiO_3_ are shown in Fig. 1a–f and Figure S1–S6 (raw patterns). The result indicates reasonable agreement with JCPDS 76–213 for BaSO_4_. As a result, it has been crystallized as an orthorhombic crystal. Stars in this pattern indicate diffraction peaks that can be indexed quite well by a tetragonal BaTiO_3_ cell with JCPDS 812203. Cu, Fe, S, N, and sucrose did not have a significant effect on the crystal structures. The EDX results confirm their presence in related samples.

**Figure 2:**
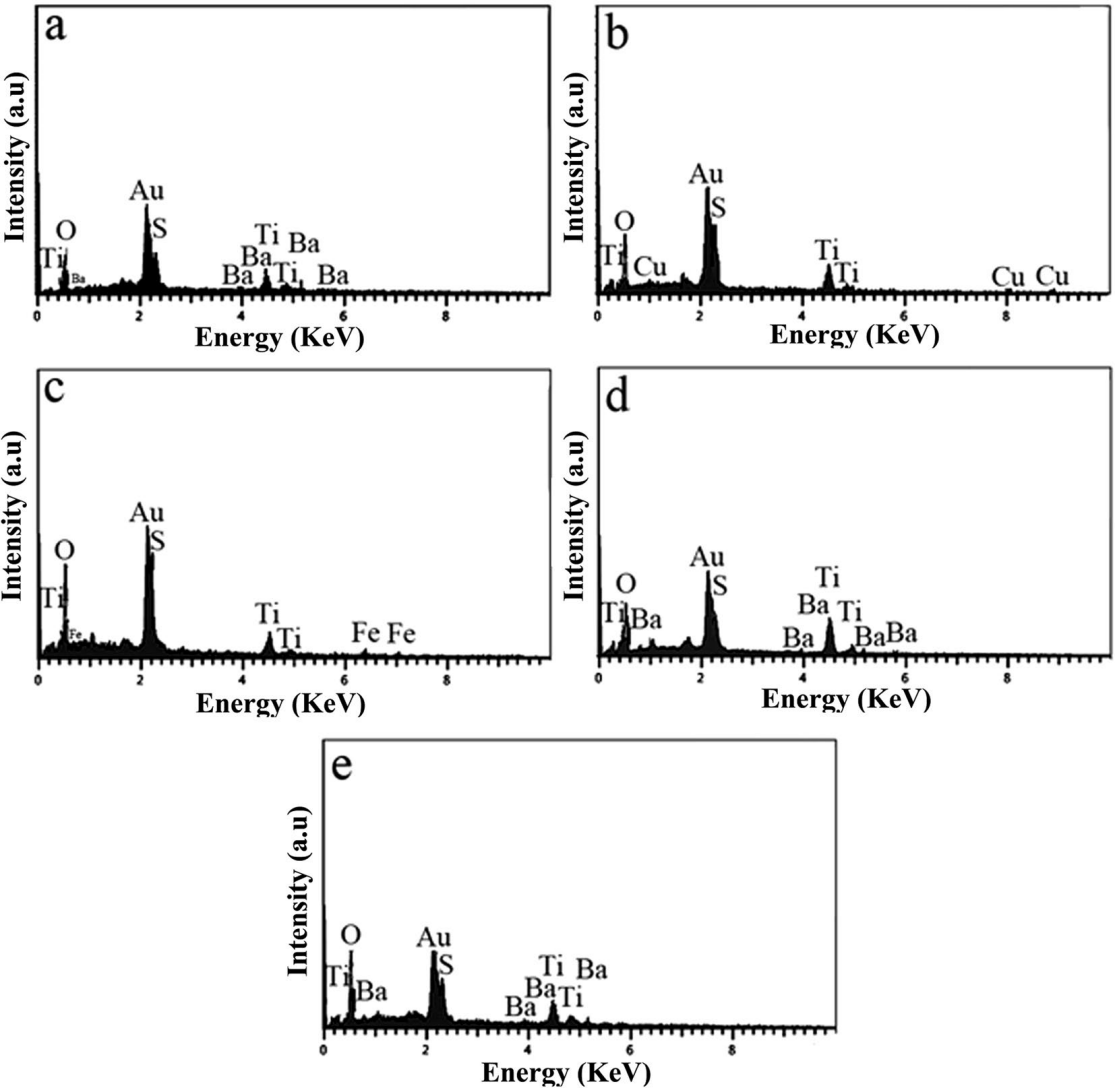
EDX for (**a**) BaSO_4_–BaTiO_3_, (**b**) Cu–BaSO_4_–BaTiO_3_, (**c**) Fe–BaSO_4_–BaTiO_3_, (**d**) N–BaSO_4_–BaTiO_3_ and (**e**) S–BaSO_4_–BaTiO_3._

The EDX for BaSO_4_–BaTiO_3_, Cu–BaSO_4_–BaTiO_3_, Fe–BaSO_4_–BaTiO_3_, N–BaSO_4_–BaTiO_3_, and S–BaSO_4_–BaTiO_3_ was demonstrated in Fig. 2a–e. Figure 2a shows the sample containing Ba, S, Ti, and O elements that could be assigned to the BaSO_4_–BaTiO_3_ composite. By adding CuSO_4_ in the synthesis step, the copper element appeared in the EDX. This indicated that Cu successfully doped into the composite (Fig. 2b). Also, the EDX results approved that Cu has been doped into the BaTiO_3_ (in BaSO_4_–BaTiO_3_ composite) structure (Figure S1 in supporting information). As SEM and EDX in Figure S1 show, the smaller particles contain Cu and Ti, Ba, and O while the big particles mainly contain Ba, S and O. Therefore, the big particles could be related to the BaSO_4_ and the smaller particles form Cu doped BaTiO_3_. Figure 2c demonstrates the EDX of Fe–BaSO_4_–BaTiO_3_. EDX approved the presence of Ba, Ti, S, O, and Fe. As Figure S2 shows, the same scenario happened and the smaller particles were Fe-doped BaTiO_3_ while the bigger particles were BaSO_4_ in the BaSO_4_–BaTiO_3_ composite system. The EDX of N–BaSO_4_–BaTiO_3_ was shown in Fig. 2d. According to this result, nitrogen does not appear in the related EDX. This could happen because of two reasons. First, nitrogen does not dope into BaTiO_3_. Second, the amount of nitrogen is less than 1 W %, therefore it does not appear in EDX. EDX of S–BaSO_4_–BaTiO_3_ was presented in Fig. 2e. The EDX showed the presence of Ba, Ti, O, and S. In this case, the small particles belong to the BaTiO_3_ again. As can be seen, it contains sulfur. It means sulfur is successfully doped in BaTiO_3_ in the BaSO_4_–BaTiO_3_ system (Figure S3).

**Figure 3:**
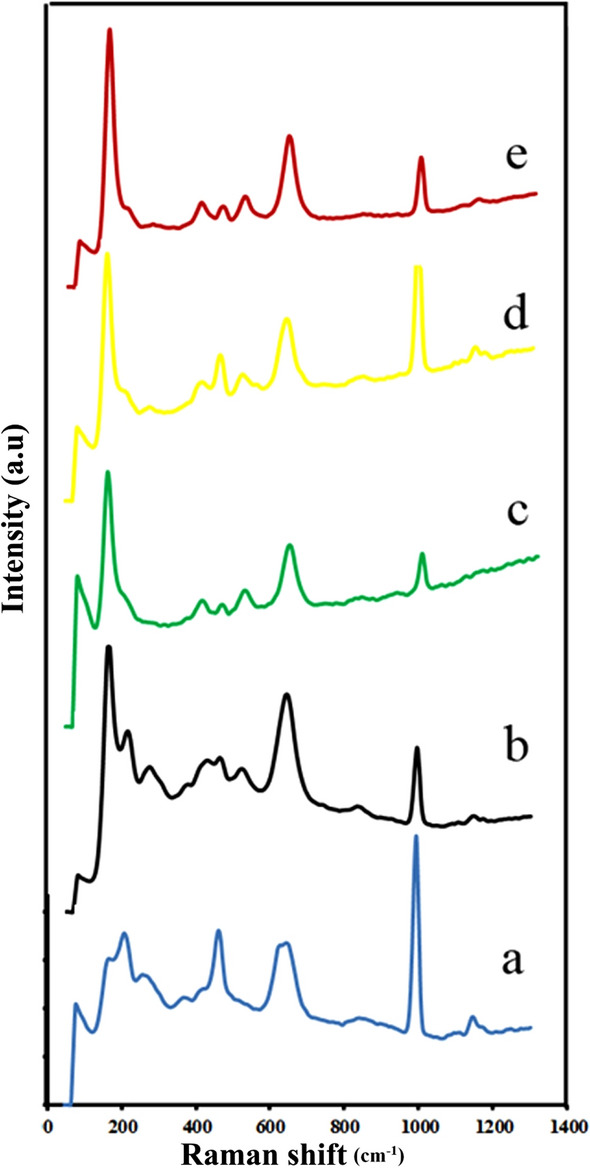
Raman spectra of (**a**) BaSO_4_–BaTiO_3_, (**b**) Cu–BaSO_4_–BaTiO_3_, (**c**) Fe–BaSO_4_–BaTiO_3_, (**d**) S– BaSO_4_–BaTiO_3_, (**e**) N–BaSO_4_–BaTiO_3_, and (**f**) S–BaSO_4_–BaTiO_3_.

Raman spectroscopy is an appropriate method for exploring chemical bonding and the solid-state structure of crystals. Dopants can also be detected using Raman spectroscopy in host-crystal lattices^34–36^. Raman spectra of the composite BaSO_4_–BaTiO_3_, when BaTiO_3_ was not doped, are shown in Fig. 3a. Figure 3b illustrates a Raman spectrum of the composite Cu–BaSO_4_–BaTiO_3_. As a consequence of the doping of Cu in the BaTiO_3_ crystal, the peak at 403 cm^−1^ is associated with the peak at 403 cm^−1^. Fe doping resulted in the disappearance of the peak at 1138 cm^−1^ and the appearance of new Raman shifts at 514 cm^−1^ and 145 cm^−1^ (Fig. 3c). As a result of the addition of N to BaTiO_3_, new Raman shifts are observed around 514 cm^−1^ and 1171 cm^−1^ (Fig. 3d). A new Raman shift was observed at 514 cm^−1^ after BaTiO_3_ was doped with S as a dopant (Fig. 3e).

**Figure 4:**
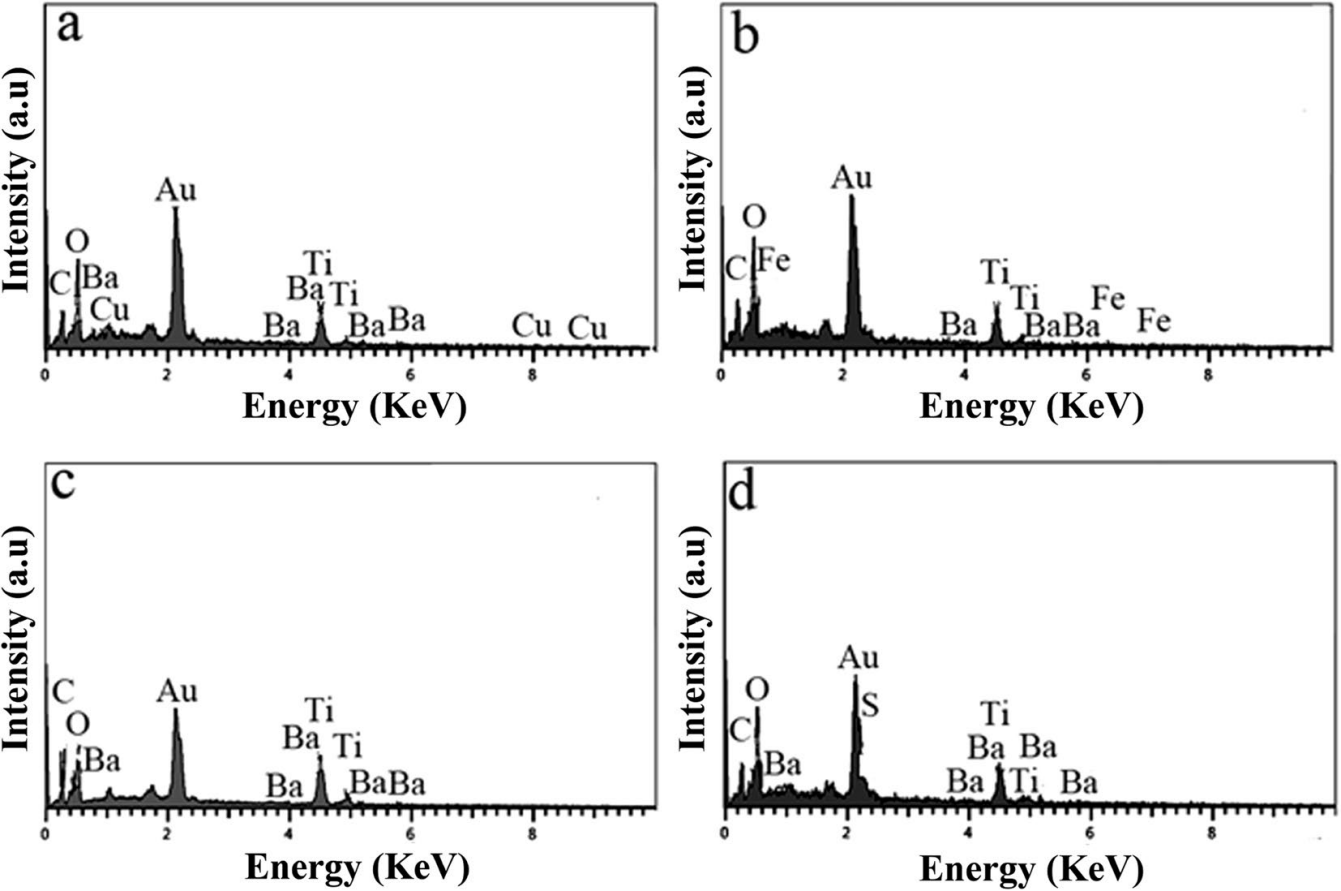
EDX of (**a**) Cu–BaSO_4_–BaTiO_3_, (**b**) Fe–BaSO_4_–BaTiO_3_, (**c**) N–BaSO_4_–BaTiO_3_ and (**d**) S–BaSO_4_–BaTiO_3_ modified by sucrose.

Figure 4a–d shows the EDX of Cu–BaSO_4_–BaTiO_3_, Fe–BaSO_4_–BaTiO_3_, N–BaSO_4_–BaTiO_3_, and S–BaSO_4_–BaTiO_3_ modified by sucrose. Figure 4a illustrates the EDX of Cu–BaSO_4_–BaTiO_3_ modified by sucrose. By comparing Figs. 2b and 4a, we can recognize that sucrose modifies Cu–BaSO_4_–BaTiO_3_. Besides Ba, Ti, O, S, and Cu, carbon has also appeared in the EDX of Cu–BaSO_4_–BaTiO_3_ modified by sucrose that could be assigned to the carbon of sucrose. Also, a comparison of Figs. 2c and 4b clarifies the presence of sucrose. The same peak appeared in the EDX of N–BaSO_4_–BaTiO_3_ and S–BaSO_4_–BaTiO_3_ modified by sucrose. This indicates that all nanostructures were successfully modified by sucrose (Fig. 4c, d). We applied FT-IR as more evidence.

**Figure 5:**
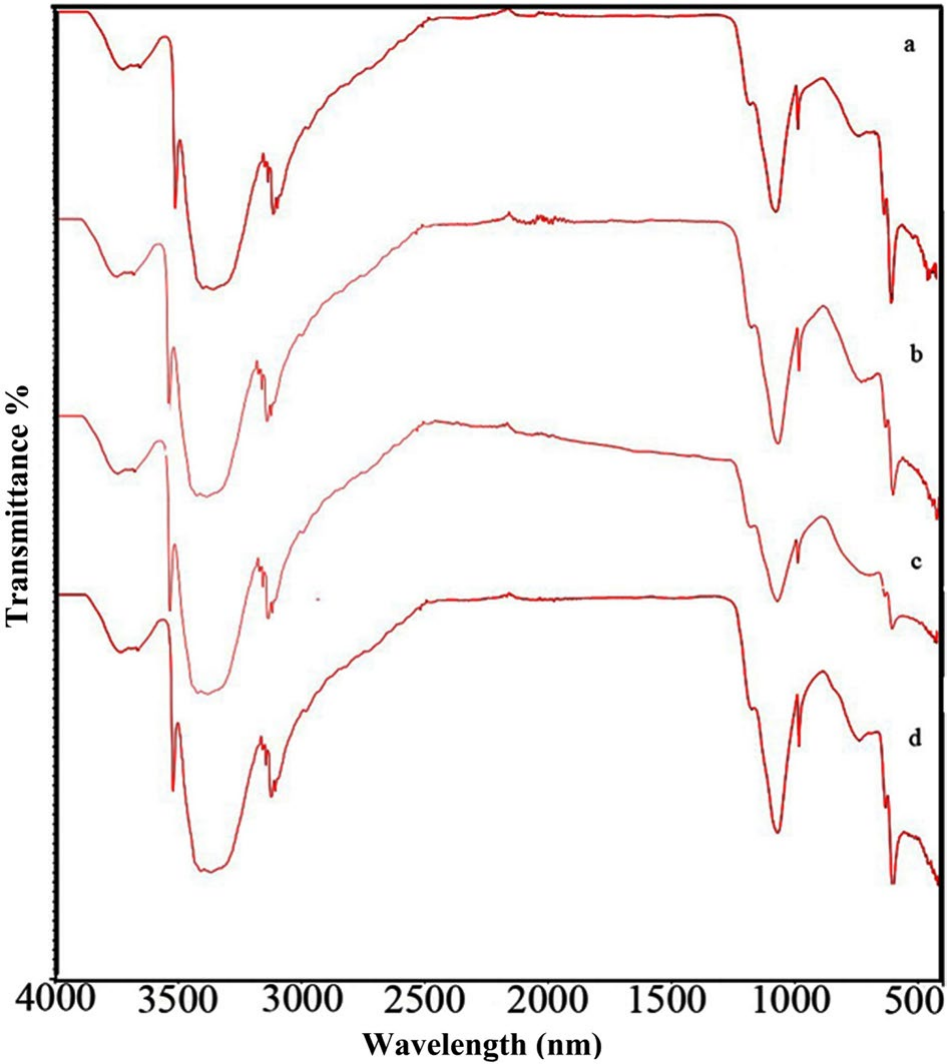
FT-IR of (**a**) Cu-BaSO_4_–BaTiO_3_, (**b**) Fe–BaSO_4_–BaTiO_3_, (**c**) N–BaSO_4_–BaTiO_3_ and (**d**) S–BaSO_4_–BaTiO_3_ modified by sucrose.

An FT-IR spectrum of CuSO_4_–BaTiO_3_, Fe–BaSO_4_–BaTiO_3_, N–BaSO_4_–BaTiO_3_, and S–BaSO_4_–BaTiO_3_ modified by sucrose is plotted in Fig. 5. In Figure S4 and Table 1, FT-IR spectra of Cu–BaSO_4_–BaTiO_3_, Fe–BaSO_4_–BaTiO_3_, N–BaSO_4_–BaTiO_3_, and S–BaSO_4_–BaTiO_3_ are also shown before the addition of sucrose. These peaks may be attributed to the sulfur–oxygen stretches found in inorganic sulfates^37^. Based on the FT-IR spectra of Figure S4 and Fig. 5, we can conclude that sucrose modifies nanostructure surfaces. The peak at 979 cm^−1^ could be associated with ring C–C stretching vibrations. The peak at ~ 1034 cm^−1^ is caused by the stretching vibration of CH_2_–OH in the C–O plane. The peak in 3000–3500 cm^−1^ could be related to sucrose's OH group.

**Table 1:** FT-IR peaks assigned to different functional groups.

Peak number	Wavenumber (cm^−1^)	Functional group
1	979	C–C stretching vibrations
2	1034	Stretching vibration of CH_2_–OH
3	1434	Asymmetric stretching of the carbonates
4	2921	CH_2_ groups
5	2854	
6	2390	
7	3000–3500	Sucrose's OH

**Figure 6:**
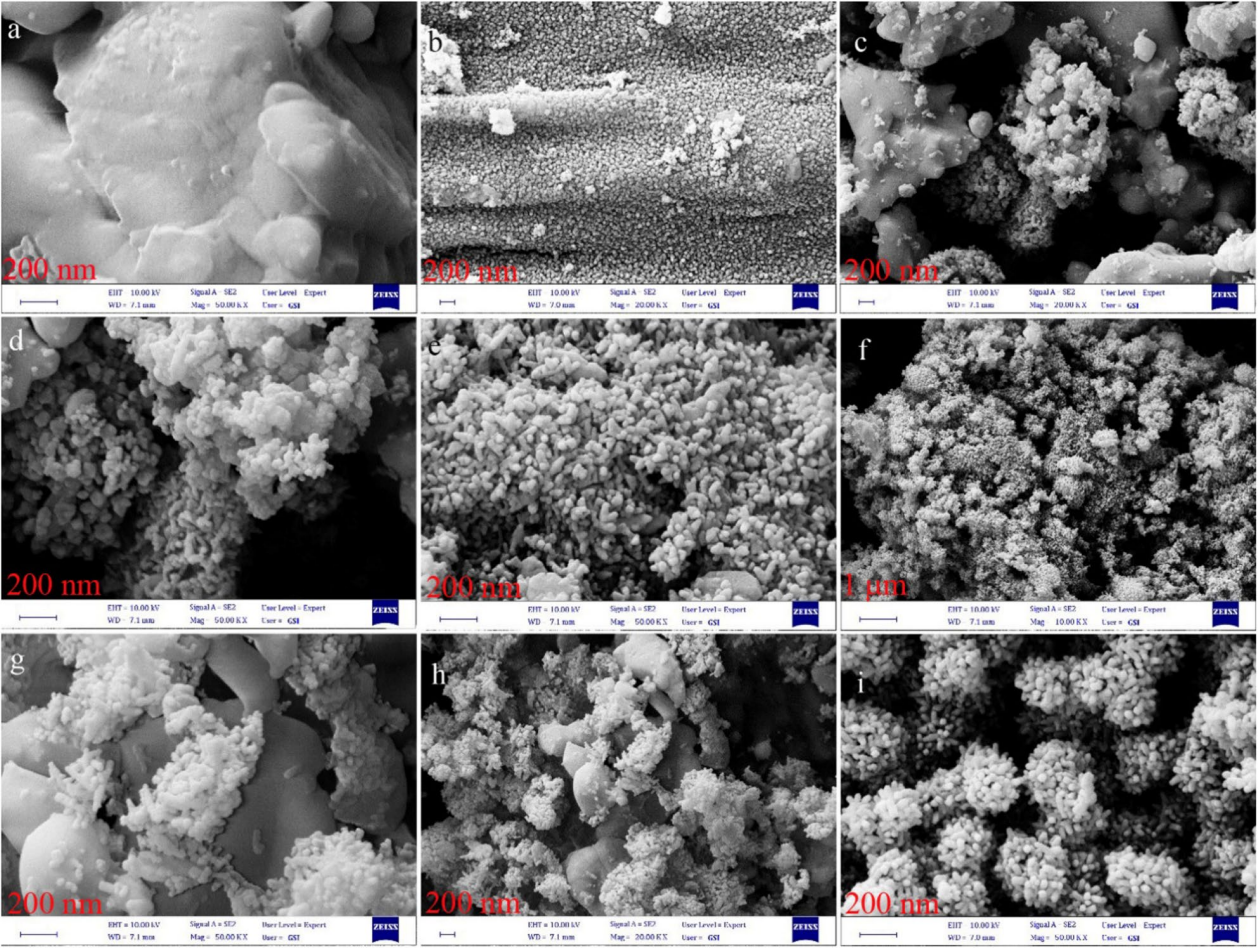
SEM images of (**a**, **b**) BaSO_4_–BaTiO_3_, (**c**, **d**) Cu–BaTiO_3_, (**e**, **f**) Fe–BaTiO_3_, (**g**, **h**) S–BaTiO_3_, and (**i**) N–BaTiO_3._

Figure 6a–i displays the SEM images of BaSO_4_–BaTiO_3_, Cu–BaSO_4_–BaTiO_3_, Fe–BaSO_4_–BaTiO_3_, S–BaSO_4_–BaTiO_3_, and N–BaSO_4_–BaTiO_3_. According to Fig. 6a, b, Figure S1, and EDX results, micro-size particles are BaSO_4_, while nanostructures form BaTiO_3_. It seems BaTiO_3_ starts to form rod-like nanostructures. In the case of Cu–BaSO_4_–BaTiO_3_ flowers like structures and microstructures could be assigned to the Cu–BaTiO_3_ and BaSO_4_, respectively (Fig. 6c, d, and Figure S1). SEM results presented in Fig. 6e, f indicate Fe–BaSO_4_–BaTiO_3_ has been assembled and formed flower-like structures. For S–BaSO_4_–BaTiO_3_, nanorod form S–BaSO_4_–BaTiO_3,_ and microstructures are BaSO_4_ (Fig. 6g, h). Figure 6i shows that very uniform flower-like structures formed when ammonia was used in the synthesis of N–BaSO_4_–BaTiO_3._

**Figure 7:**
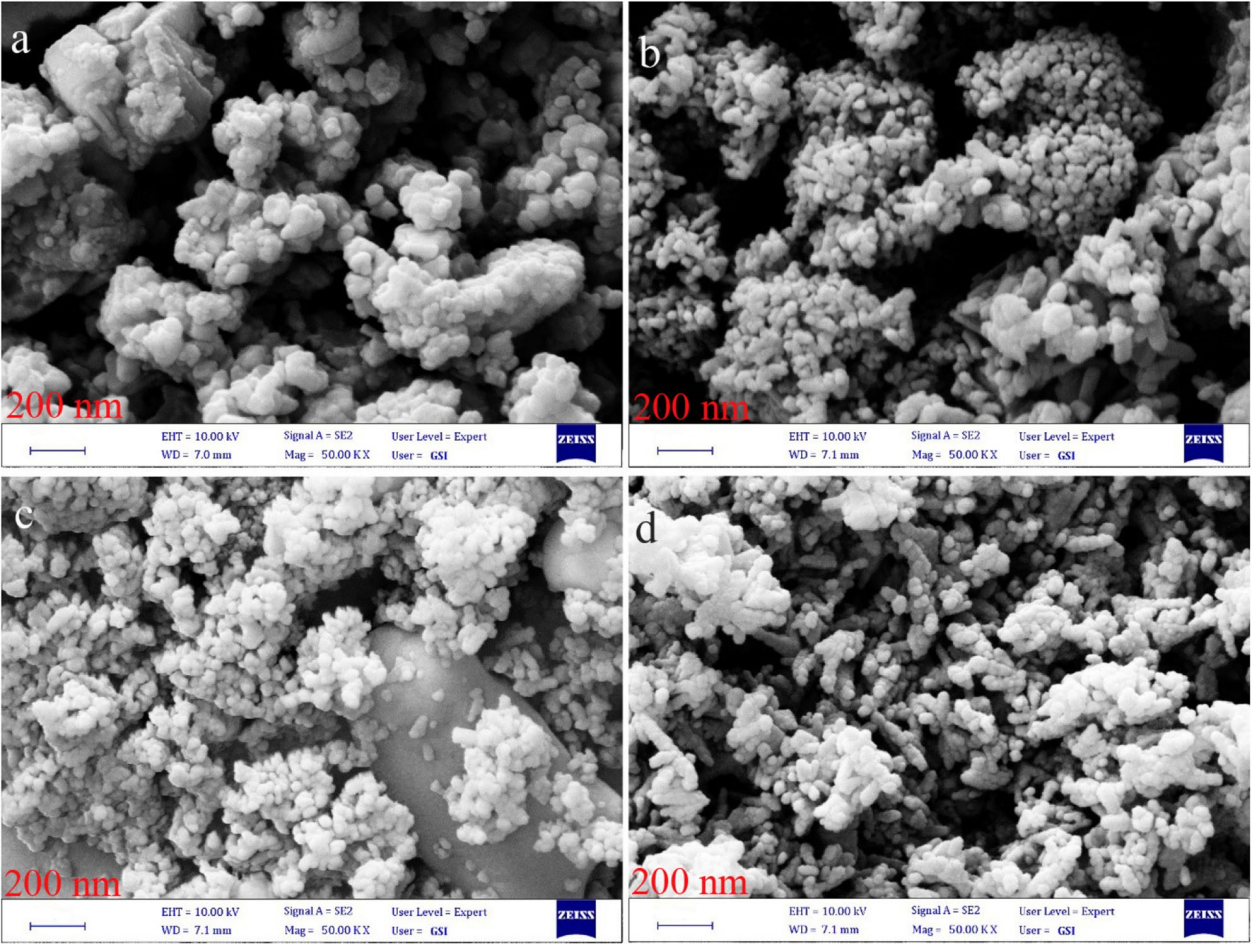
SEM images for as-prepared nanostructures including (**a**) Cu–BaSO_4_–BaTiO_3_, (**b**) Fe–BaSO_4_–BaTiO_3_, (**c**) N–BaSO_4_–BaTiO_3_ and (**d**) S–BaSO_4_–BaTiO_3_ modified by sucrose.

SEM images for as-prepared nanostructures including Cu–BaSO_4_–BaTiO_3_, Fe–BaSO_4_–BaTiO_3_, N–BaSO_4_–BaTiO_3_, and S–BaSO_4_–BaTiO_3_ modified by sucrose are summarized in Fig. 7a–d. SEM image of Cu–BaSO_4_–BaTiO_3_-sucrose is illustrated in Fig. 7a. We can figure out sucrose cover Cu–BaSO_4_–BaTiO_3_ and stuck particles by comparing it with SEM images of Cu–BaSO_4_–BaTiO_3_ (Fig. 6c, d). The same conclusion could be made by comparing SEM images of nanostructures before sucrose (Fig. 6e–i) and after modification with sucrose (Fig. 7b–d).

**Figure 8:**
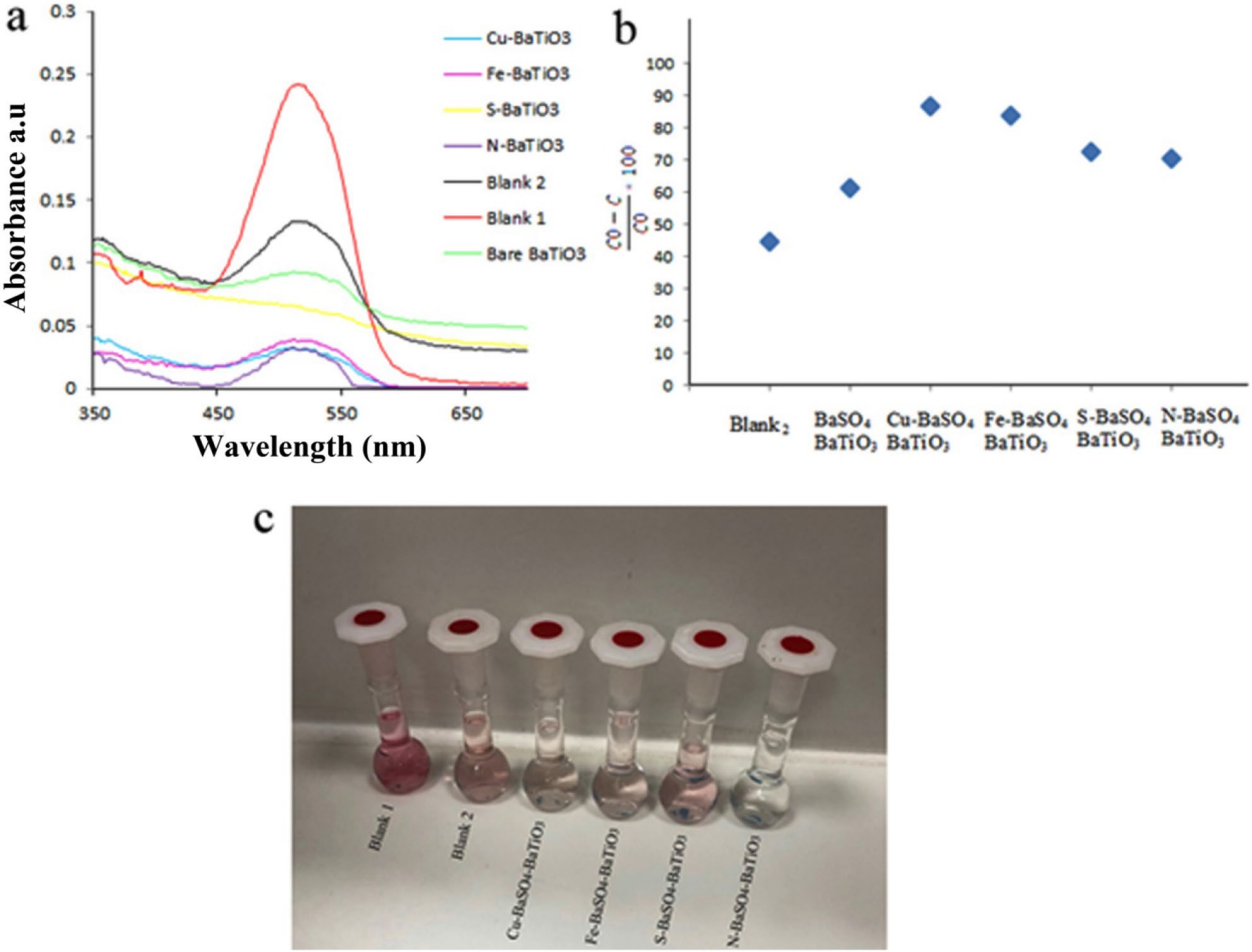
(**a**) The UV–Vis spectrum of initial AR151 (blank 1), AR151 after treat it with bare ultrasonic (blank 2), BaSO_4_–BaTiO_3_ (bare BaTiO_3_), Cu–BaSO_4_–BaTiO_3_ (Cu–BaTiO_3_), Fe–BaSO_4_–BaTiO_3_ (Fe–BaTiO_3_), S–BaSO_4_–BaTiO_3_ (S–BaTiO_3_), and N–BaSO_4_–BaTiO_3_ (N–BaTiO_3_). (**b**) Degradation efficiency by using bare ultrasonic (blank 2), BaSO_4_–BaTiO_3_ (bare BaTiO_3_), Cu–BaSO_4_–BaTiO_3_ (Cu–BaTiO_3_), Fe–BaSO_4_–BaTiO_3_ (Fe–BaTiO_3_), S–BaSO_4_–BaTiO_3_ (S–BaTiO_3_), and N–BaSO_4_–BaTiO_3_ (N–BaTiO_3_). (**c**) Show the photo of blank 1, blank 2, Cu––BaSO_4_–BaTiO_3_, Fe–BaSO_4_–BaTiO_3_, N–BaSO_4_–BaTiO_3_ and S–BaSO_4_–BaTiO_3_ after 90 min vibration and centrifuge.

**Figure 9:**
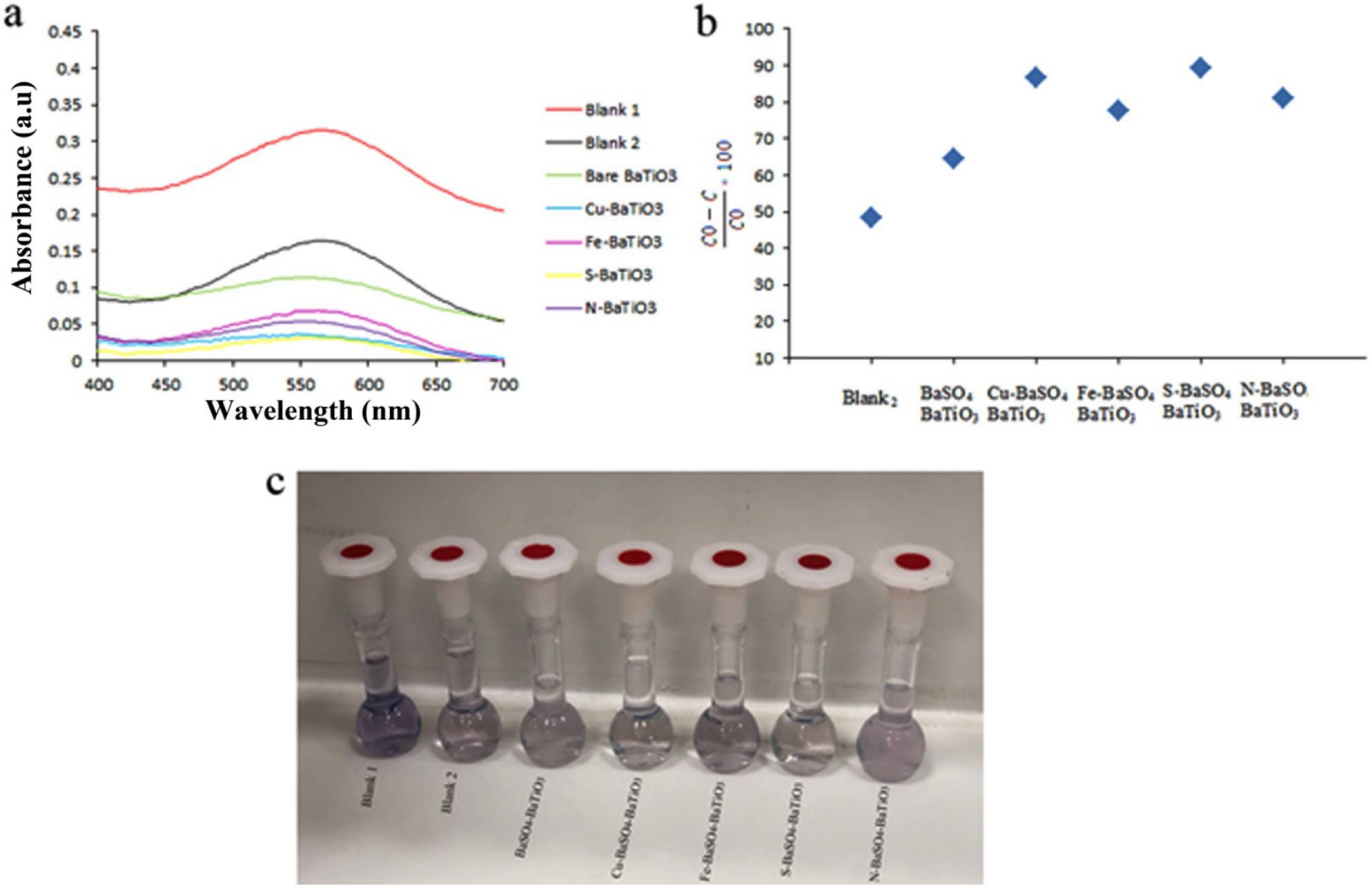
(**a**) The UV–Vis spectrum of initial AB113 (blank 1), AB113 after treat it with bare ultrasonic (blank 2), BaSO_4_–BaTiO_3_ (bare BaTiO_3_), Cu–BaSO_4_–BaTiO_3_ (Cu–BaTiO_3_), Fe–BaSO_4_–BaTiO_3_ (Fe–BaTiO_3_), S–BaSO_4_–BaTiO_3_ (S–BaTiO_3_), and N–BaSO_4_–BaTiO_3_ (N–BaTiO_3_). (**b**) Degradation efficiency by using bare ultrasonic (blank 2), BaSO_4_–BaTiO_3_ (bare BaTiO_3_), Cu–BaSO_4_–BaTiO_3_ (Cu–BaTiO_3_), Fe–BaSO_4_–BaTiO_3_ (Fe–BaTiO_3_), S–BaSO_4_–BaTiO_3_ (S–BaTiO_3_), and N–BaSO_4_–BaTiO_3_ (N–BaTiO_3_). (**c**) Show the photo of blank 1, blank 2, BaSO_4_–BaTiO_3_, Cu–BaSO_4_–BaTiO_3_, Fe–BaSO_4_–BaTiO_3_, N–BaSO_4_–BaTiO_3_ and S–BaSO_4_–BaTiO_3_ after 90 min vibration and centrifuge.

Piezocatalytic activity of Pure BaSO_4_, BaSO_4_–BaTiO_3_, Cu–BaSO_4_–BaTiO_3_, Fe–BaSO_4_–BaTiO_3_, S–BaSO_4_–BaTiO_3_, N–BaSO_4_–BaTiO_3_, and m-BaTiO_3_ was evaluated by degradation of AR151 and AB113 under external mechanical force (ultrasonic vibration). Figure 8a–c and Table 2 present results for the piezocatalytic degradation of AR151 by ultrasonic vibration without catalyst, BaSO_4_–BaTiO_3_, Cu–BaSO_4_–BaTiO_3_, Fe–BaSO_4_–BaTiO_3_, S–BaSO_4_–BaTiO_3_, and N–BaSO_4_–BaTiO_3_. Also, the degradation efficiency of Pure BaSO_4_ for AR1 and AB113 is presented in Figure S5. According to Figure S5, Pure BaSO_4_ degrade 56.7% and 60.9% of AR151 and AB113 during 90 min ultrasonic. The red curve in Fig. 8a shows the UV–Vis spectrum of the initial AR151 solution. The black curve represents the spectrum for AR151 when it was vibrated by 100 W ultrasonic without a catalyst. We label it as Blank 2. As can see from Fig. 8 and Table 2, 44.8% of AR151 was degraded during 90 min by bare ultrasonic waves with 2 on:2 off the pulse. When BaSO_4_–BaTiO_3_ was added, degradation increased to 61.3% for AR151 (green curve). N–BaSO_4_–BaTiO_3_ and S–BaSO_4_–BaTiO_3_ degrade 88.5 and 72.6% of AR151, respectively. In the case of AR151, using the S dopant does not show a significant effect on the piezocatalytic activity of BaSO_4_–BaTiO_3_, while using N, Cu^+2^, and Fe^+3^ as dopant significantly improve decontamination yield. Decontamination yields of 85.9% and 83.8% for doped BaTiO_3_ with Cu^+2^ and Fe^+3^ for AR151 were achieved, respectively. As the results in Fig. 8a–c and Table 2 show, Cu–BaSO_4_–BaTiO_3_ shows promising degradation efficiency compare to the –BaSO_4_–BaTiO_3_. It seems to replace A and B cations in perovskite structure with the general formula of ABX_3_ shows more effect on the piezocatalytic activity of composite. Besides BaSO_4_–BaTiO_3_, Cu–BaSO_4_–BaTiO_3_, Fe–BaSO_4_–BaTiO_3_, S–BaSO_4_–BaTiO_3_, and N–BaSO_4_–BaTiO_3_ were applied to treat water containing AB113, Fig. 9a–c and Table 3 reveal related results. Related results show that 48.3% of AB113 was degraded during 90 min ultrasonic vibration without the catalyst. Adding BaSO_4_–BaTiO_3_ as a piezocatalyst leads to an increased piezocatalytic degradation of AB113 to 64.4%. By changing the piezocatalyst to Cu–BaSO_4_–BaTiO_3_ degradation efficiency increased to 86.7%. However, by changing the catalyst to Fe–BaSO_4_–BaTiO_3_ degradation efficiency of 77.6% was achieved. In the case of using the BaSO_4_–BaTiO_3_ series catalyst, S–BaSO_4_–BaTiO_3_ shows the highest efficiency. It degrades 89.2% of AB113 during 90 min ultrasonic. Finally, we examine N–BaSO_4_–BaTiO_3_ as a piezocatalyst in the same operating condition. A decontamination efficiency of 80.9% was achieved. Results approve that dopants could show dramatically effect on piezocatalytic activity. The origin of these improvements could be the following reasons: (I) dopant could act as a shallow level acceptor in BaTiO_3_ and can significantly reduce the piezoelectric potential screening effect^25–29,38^. (II) Depending on the radius of the dopant, it could create an increased strain while replacing the Ba or Ti in the BaTiO_3_ lattice, thus leading to an increase in the piezoelectric coefficient^30–32^. (III): dopant could increase electrical resistivity and reduce charge leakage^33^.

**Table 2:** Piezoelectric catalytic capability of Pure BaSO_4_, BaSO_4_–BaTiO_3_, Cu–BaSO_4_–BaTiO_3_, Fe–BaSO_4_–BaTiO_3_, S–BaSO_4_–BaTiO_3_, N–BaSO_4_–BaTiO_3_ nanostructure for degradation of AR151.

Catalyst	Ultrasonic time (min)	Pulse: on: off (s)	Power (W)	Decontamination efficiency (%)
W/O catalyst (Blank 2)	90	2:2	100	44.8
Pure BaSO_4_	90	2:2	100	56.7
BaSO_4_–BaTiO_3_	90	2:2	100	61.3
N–BaSO_4_–BaTiO_3_	90	2:2	100	88.5
S–BaSO_4_–BaTiO_3_	90	2:2	100	72.6
Cu–BaSO_4_–BaTiO_3_	90	2:2	100	85.9
Fe–BaSO_4_–BaTiO_3_	90	2:2	100	83.8

**Table 3:** Piezoelectric catalytic capability of Pure BaSO_4_, BaSO_4_–BaTiO_3_, Cu–BaSO_4_–BaTiO_3_, Fe–BaSO_4_–BaTiO_3_, S–BaSO_4_–BaTiO_3_, N–BaSO_4_–BaTiO_3_ nanostructure for degradation of AB113.

Catalyst	Ultrasonic time (min)	Pulse: on: off (s)	Power (W)	Decontamination efficiency (%)
W/O catalyst (Blank 2)	90	2:2	100	48.3
Pure BaSO_4_	90	2:2	100	60.9
BaSO_4_–BaTiO_3_	90	2:2	100	64.4
N–BaSO_4_–BaTiO_3_	90	2:2	100	80.9
S–BaSO_4_–BaTiO_3_	90	2:2	100	89.2
Cu–BaSO_4_–BaTiO_3_	90	2:2	100	86.7
Fe–BaSO_4_–BaTiO_3_	90	2:2	100	77.6

In another strategy, we modified doped BaTiO_3_ (in the BaSO_4_–BaTiO_3_ system) with sucrose to improve its piezocatlytic activity. Sucrose is a natural piezocatalytic material, therefore it could improve piezocatlytic activity^37,39^.

**Figure 10:**
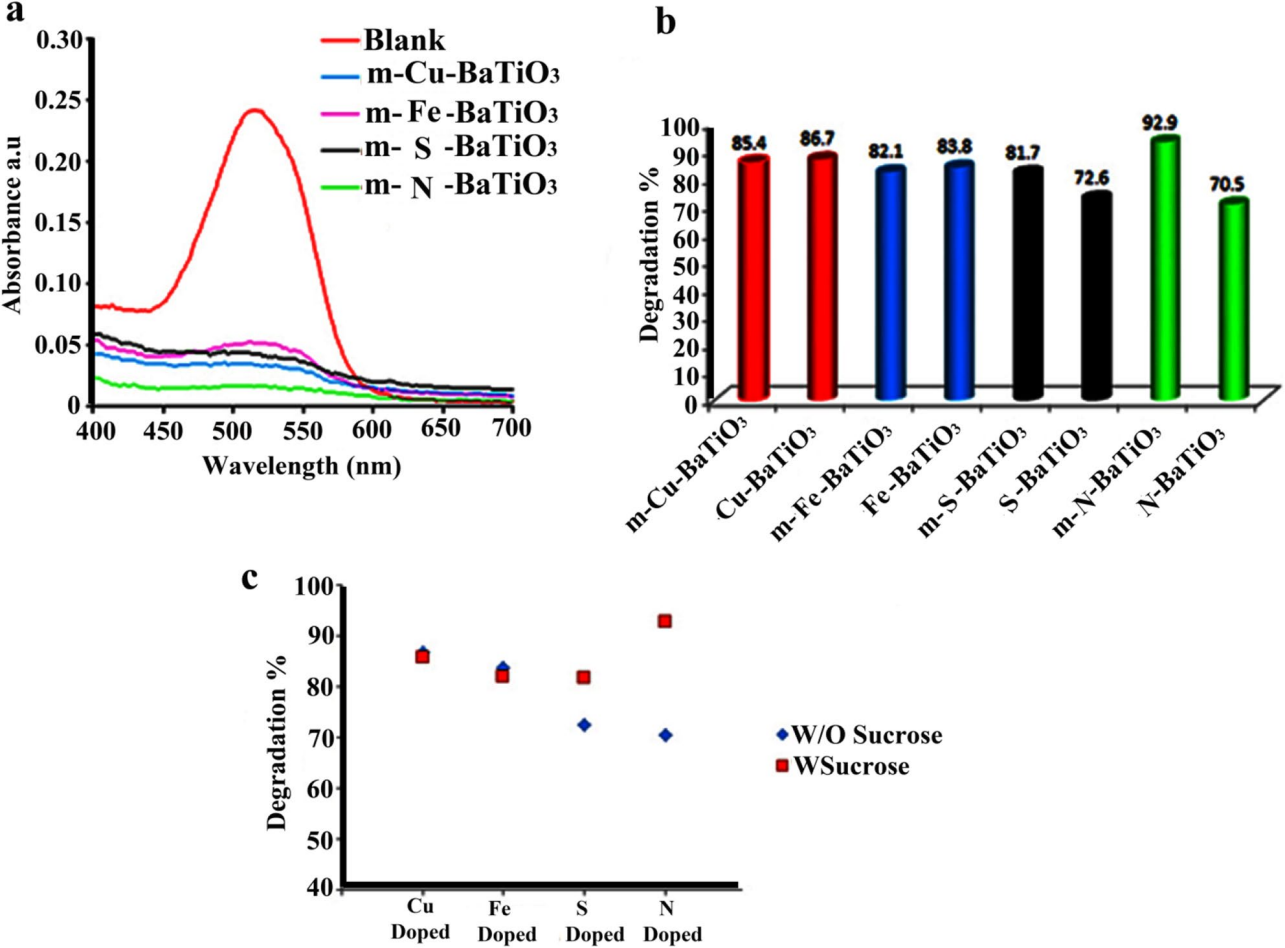
Effect of sucrose on piezocatalytic activity of doped BaTiO_3_ to degrade AR151. (**A**) related UV–Vis spectra, (**b**) related degradation efficiency, and (**c**) compare degradation for sucrose and without sucrose.

Figure 10a–c and Table 4 show the effect of sucrose on the piezocatalytic activity of doped BaSO_4_–BaTiO_3_ to degrade AR151. We labeled the modified samples as follows: m-Cu–BaSO_4_–BaTiO_3_ (for Cu doped BaTiO_3_ modified by sucrose), m-Fe–BaSO_4_–BaTiO_3_ (for Fe doped BaTiO_3_ modified by sucrose), m-S–BaSO_4_–BaTiO_3_ (for S doped BaTiO_3_ modified by sucrose), and m-N–BaSO_4_–BaTiO_3_ (for N doped BaTiO_3_ modified by sucrose). M-Cu–BaSO_4_–BaTiO_3_ degraded 85.9% of AR151 during 90 min ultrasonic vibration, while Cu–BaTiO_3_ degraded 86.7% of AR151 in the same vibration time. Fe-doped BaSO_4_–BaTiO_3_ and m-Fe–BaSO_4_–BaTiO_3_ almost showed the same degradation efficiency, Fe–BaSO_4_–BaTiO_3_ and m-Fe–BaSO_4_–BaTiO_3_ degraded 83.8 and 82.1% of AR151, respectively. S-doped BaSO_4_–BaTiO_3_ modified by sucrose showed better performance compare to the S-BaSO_4_–BaTiO_3_. S-BaSO_4_–BaTiO_3_ showed 72.6% degradation efficiency, while m-S–BaSO_4_–BaTiO_3_ degraded 81.7% of AR151. Finally, N-doped BaSO_4_–BaTiO_3_ was modified by sucrose. As can be seen, m-N–BaSO_4_–BaTiO_3_ shows higher degradation efficiency. M-N–BaSO_4_–BaTiO_3_ degraded 92.9% of AR151 which was much higher than the degradation yield for N–BaSO_4_–BaTiO_3_ (88.5%).

**Table 4:** Effect of sucrose on piezocatalytic activity of doped BaTiO_3_ to degrade AR151.

Catalyst	Ultrasonic time (min)	Pulse: on: off (s)	Power (W)	Decontamination efficiency (%)
m-N–BaSO_4_–BaTiO_3_	90	2:2	100	92.9
m-S–BaSO_4_–BaTiO_3_	90	2:2	100	81.7
m-Cu–BaSO_4_–BaTiO_3_	90	2:2	100	85.9
m-Fe–BaSO_4_–BaTiO_3_	90	2:2	100	82.1

**Figure 11:**
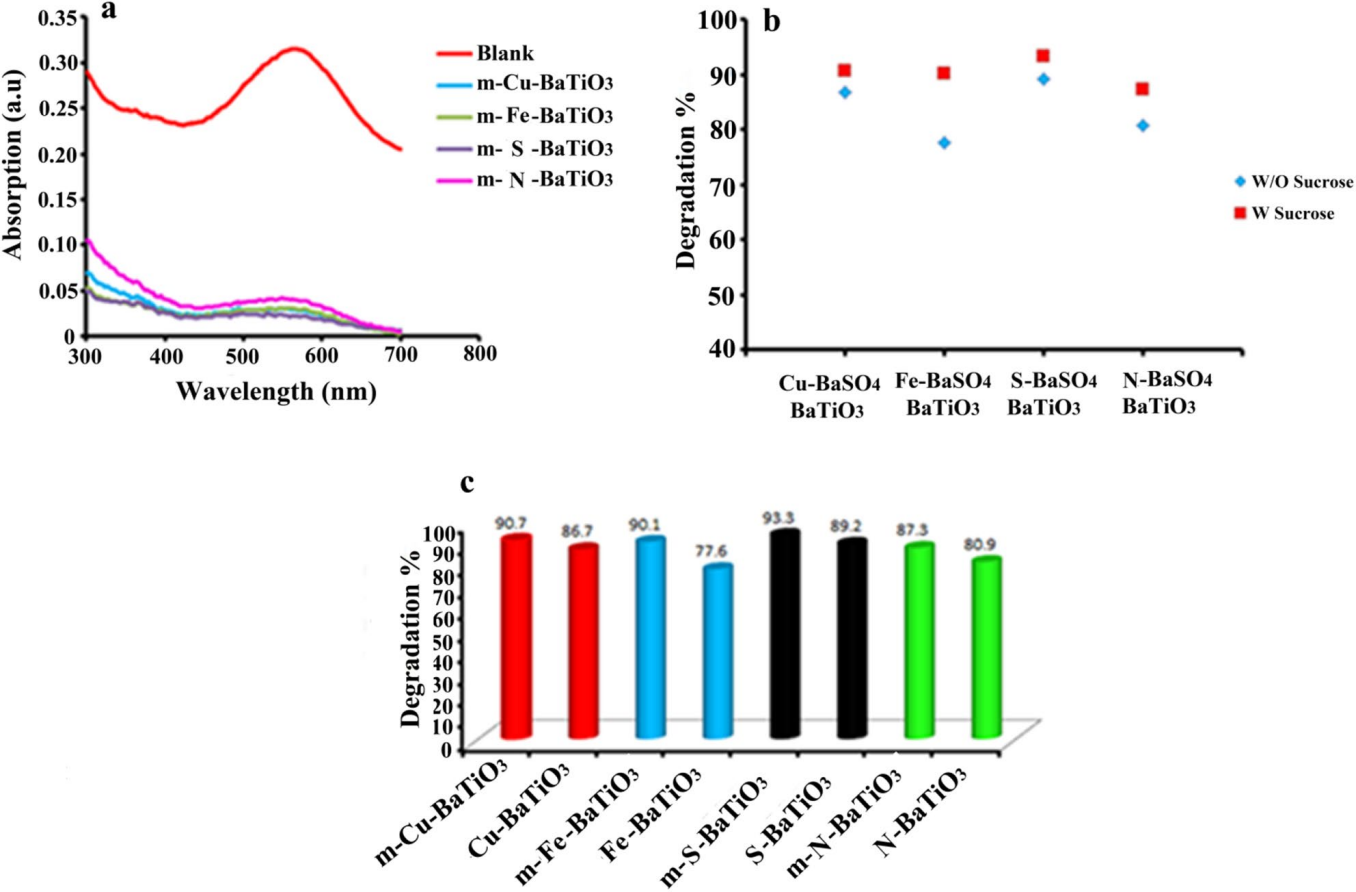
Effect of sucrose on piezocatalytic activity of doped BaTiO_3_ to degrade AB113. (**A**) related UV–Vis spectra, (**b**) compare degradation for sucrose and without sucrose, and (**c**) related degradation efficiency with sucrose.

We repeated these tests for degradation of AB113 and summarized results in Fig. 11a–c and Table 5. Cu–BaSO_4_–BaTiO_3_ degrades 86.7% of AB113 under ultrasonic waves with 100 W in power for 90 min. By displacing Cu ions with Ba in the BaTiO_3_ lattice, the smaller ionic radius results in the Cu–O bonds rotating more easily in the direction of the applied field. Thus, produces a larger piezoelectric effect and enhances the electromechanical responses. The smaller ionic radius of Cu can also result in a smaller defensive force between the ions and produces a larger displacement of Cu under stress. Therefore, when the same amount of mechanical force was applied, the dipole moment induced in Cu–BaSO_4_–BaTiO_3_ would be larger and a higher piezoelectric constant would be obtained^40–42^. By modifying it with sucrose the degradation efficiency for AB113 was increased to 90.7%. M-Fe–BaSO_4_–BaTiO_3_ decontaminates about 90.1% of AB113, while Fe–BaSO_4_–BaTiO_3_ degrades about 77.6% of AB113. Comparing the decomposition efficiency of S-doped BaSO_4_–BaTiO_3_ and S-doped BaSO_4_–BaTiO_3_ modified by sucrose showed higher decomposition efficiency for S-doped BaSO_4_–BaTiO_3_ modified by sucrose (degradation efficiency of 89.2 and 93.3% was achieved, respectively). N–BaSO_4_–BaTiO_3_ and m-N–BaSO_4_–BaTiO_3_ degraded 80.9 and 87.3% of AB113, respectively. Results showed that both dopants type and pollutants affect degradation efficiency. For example in the decontamination of AR151, Cu–BaSO_4_–BaTiO_3_ showed the highest piezocatalytic activity, while S–BaTiO_3_ showed the highest performance in decontamination AB113. Results also showed that sucrose generally could improve the piezocatalytic activity of BaSO_4_–BaTiO_3._

**Table 5:** Effect of sucrose on piezocatalytic activity of doped BaTiO_3_ to degrade AB113.

Catalyst	Ultrasonic time (min)	Pulse: on: off (s)	Power (W)	Decontamination efficiency (%)
m-N–BaSO_4_–BaTiO_3_	90	2:2	100	87.3
m-S–BaSO_4_–BaTiO_3_	90	2:2	100	93.3
m-Cu–BaSO_4_–BaTiO_3_	90	2:2	100	90.7
m-Fe–BaSO_4_–BaTiO_3_	90	2:2	100	90.1

### Study the effect of ultrasonic power and pulse on piezocatalytic degradation efficiency of AB113

**Figure 12:**
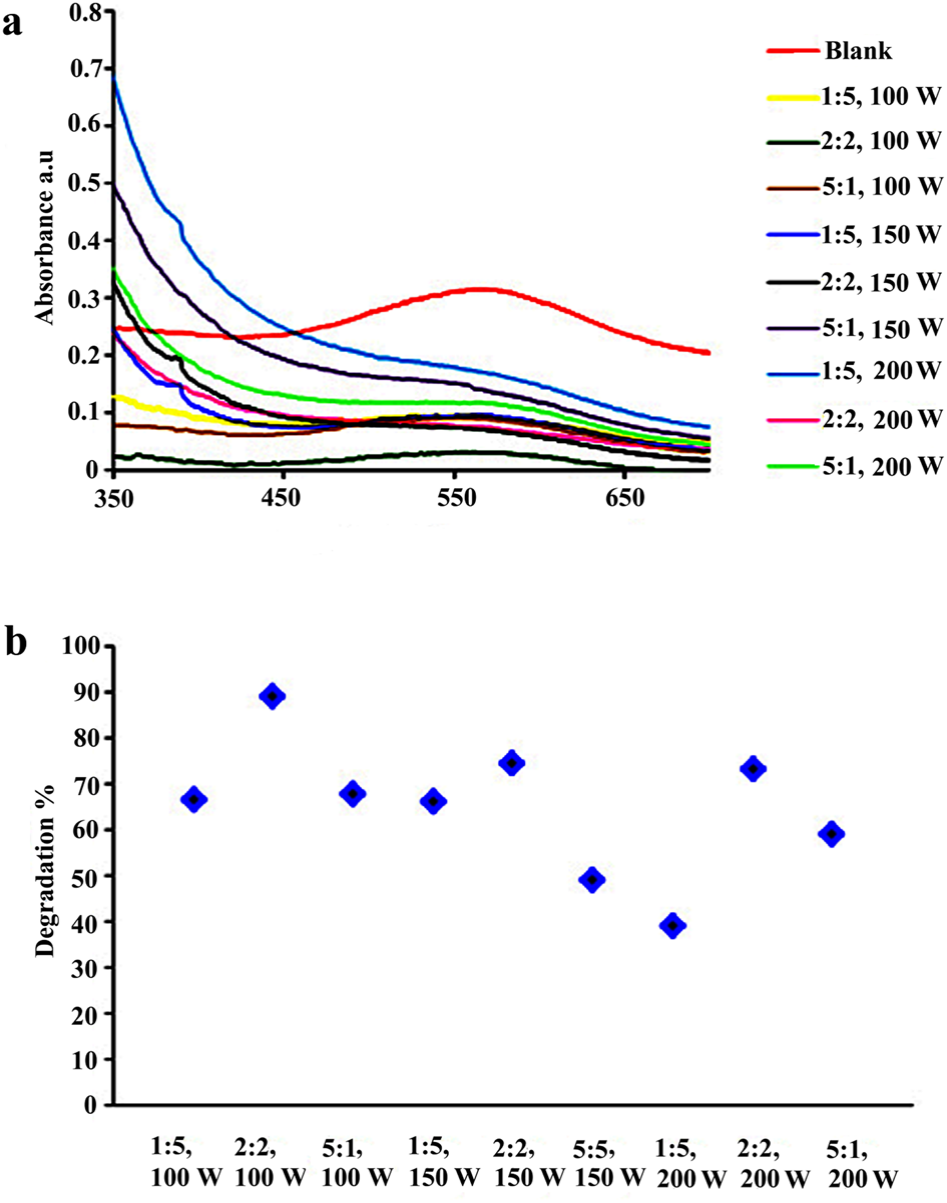
Effect of ultrasonic pulse and power on decontamination of AB113. S–BaSO_4_–BaTiO_3_ was used as the piezocatalyst: (**a**) related spectrum and (**b**) degradation results.

As a piezocatalyst, S–BaSO_4_–BaTiO_3_ was used to study the effects of ultrasonic power and pulse. Three power levels, including 100, 150, and 200 W, and three pulse rates, including 1:5, 2:2, and 5:1 s on–off, were chosen. The related spectra are shown in Fig. 12a, and the related decontamination efficiency is shown in Fig. 12b. According to the results, a pulse with 2 s on and 2 s off showed the highest decontamination efficiency for all ultrasonic powers. To produce more active radicals and exhibit piezoelectricity again, piezocatalysts need time to return to the ground state. As the ground state, we define it as the state in which positive and negative charges are symmetrically dispersed. The Piezocatalyst has enough time to return to the ground state when the pulse is 2:2 (on–off). Thus, can produce more radicals in the next vibration pulse and shows a higher degradation efficiency. As a result of increasing the power, degradation efficiency decreased. As the ultrasonic power increases, the temperature of the reaction will also rise. Our previous study showed that increasing temperature resulted in a decrease in decontamination yield because dye degradation by piezo is exothermic^43^.

### Piezocatalytic Mechanism

**Figure 13:**
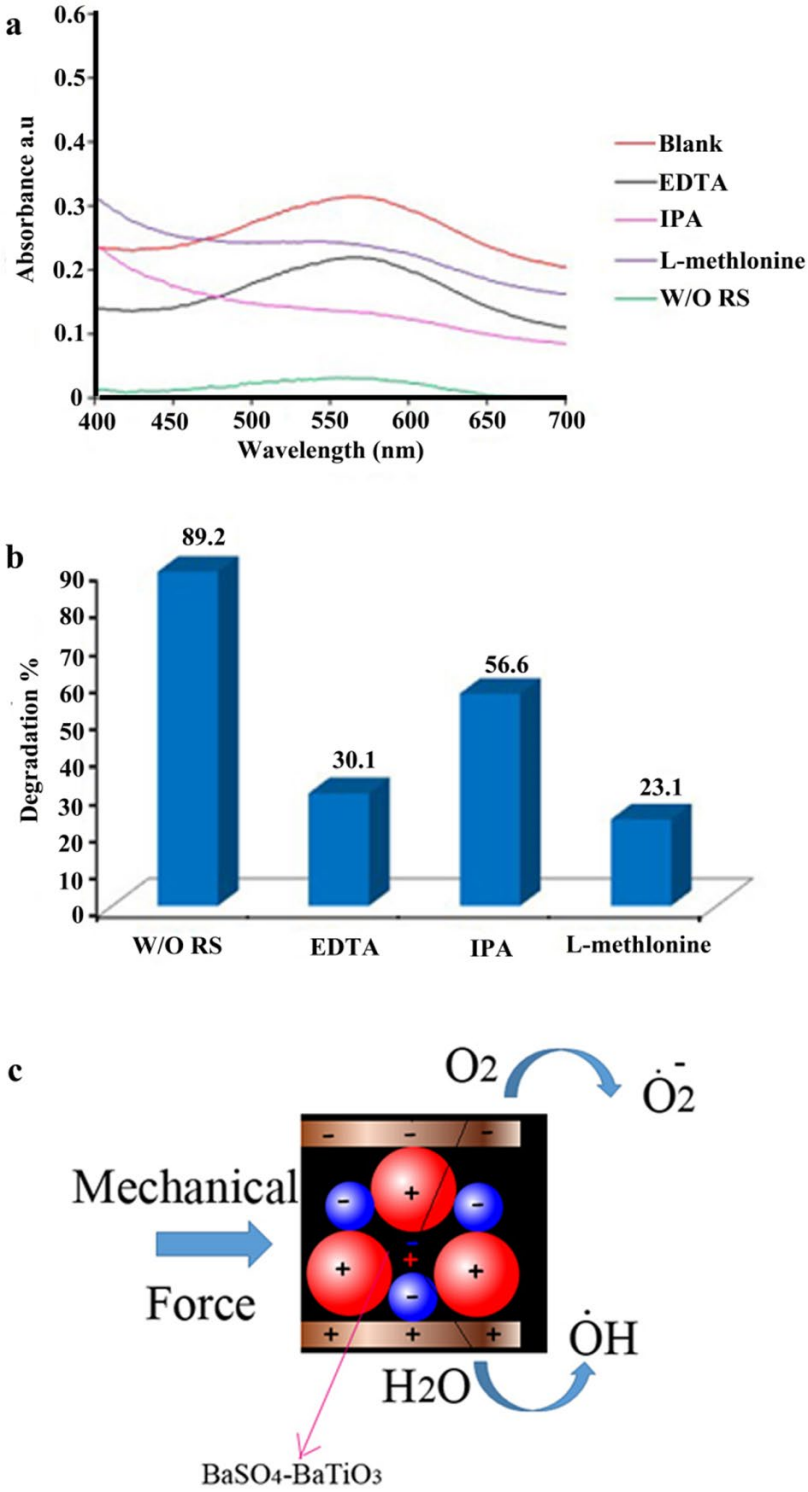
Possible mechanism for decontamination of AB113 by using a piezocatalyst. EDTA, IPA, and L-methionine as the hole (h^+^), hydroxyl radical (·OH), and peroxide radical (O2·^−2^) scavengers, respectively. (**a**) Related spectrum, (**b**) related degradation efficiency, and (**c**) schematic for piezo degradation.

To recognize a possible active species in piezocatalytic degradation of pollutants EDTA, isopropanol, and L-methionine were used as the hole (h^+^), hydroxyl radical (·OH), and peroxide radicals (O_2_·^−^) scavengers, respectively. Results are summarized in Fig. 13a–c. Based on the results EDTA, isopropanol, and L-methionine significantly suppressed the piezoelectric decontamination process. By adding EDTA, L-methionine, and IPA, the decontamination efficiencies were decreased from 89.2% to 30.1%, 56.6%, and 23.1.0%, respectively. Radical trapping evaluation indicated that the piezo-generated O_2_·^−^ and holes (h^+^) played the main role in the piezoelectric decontamination of AB113^44–47^. According to Fig. 13c, by applying mechanical force (ultrasonic waves) the centers of symmetry of the charges move apart. They no longer coincide and give rise to the net charge on the surface. These positive and negative charges react with oxygen and water and produce active radical species that could degrade organic pollutants. Based on the results, the degradation process model could be as follow:

1 $$ {\text{BaSO}}_{{{\text{4 - BaTiO}}_{3} }} {\mkern 1mu} {\text{composite}} + {\text{Vibration}} \to {\text{BaSO}}_{{{\text{4 - BaTiO}}_{3} }} {\mkern 1mu} {\text{composite}}{\mkern 1mu} ({\text{e}}^{ - }  + {\text{h}}^{ + } )  $$

2 $$ {\text{e}}^{ - } + {\text{O}}_{{2}} \to {\text{O}}_{{2}}^{ - } $$

3 $$ {\text{h}}^{ + } + {\text{H}}_{{2}} {\text{O}} \to {\text{H}}^{ + } +^{ \cdot } {\text{OH}} $$

4 $$^{ \cdot } {\text{O}}_{{2}}^{ - } + {\text{dye}} \to {\text{CO}}_{{2}} + {\text{H}}_{{2}} {\text{O}} + {\text{by-products}} $$

5 $$ {\text{h}}^{ + } + {\text{dye}} \to {\text{CO}}_{{2}} + {\text{H}}_{{2}} {\text{O}} + {\text{by-products}} $$

6 $$^{ \cdot } {\text{OH}} + {\text{dye}} \to {\text{CO}}_{{2}} + {\text{H}}_{{2}} {\text{O}} + {\text{by-products}} $$

#### Reusability of piezo-catalyst

**Figure 14:**
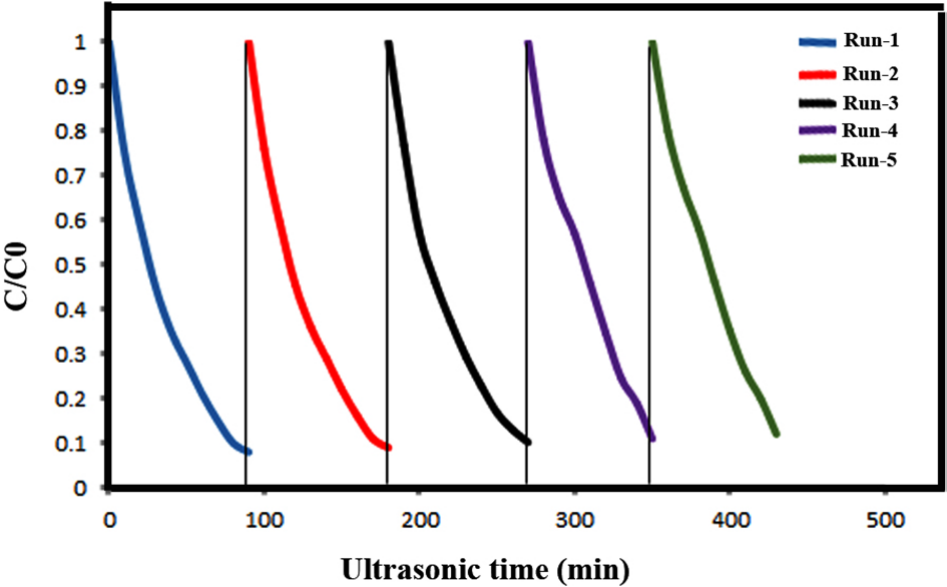
Reusability of the m-S–BaSO_4_–BaTiO_3_ in five successive experimental runs for the piezocatalytic degradation of AB113 in aqueous solution under ultrasonic irradiation.

In addition to piezo-catalytic efficiency, the reusability of a piezo-catalyst is an important factor for practical applications. Five successive piezo-catalytic experimental runs were conducted to evaluate the stability of as-prepared piezo-catalysts in operation conditions by adding recycled m-S–BaSO_4_–BaTiO_3_ nanomaterial to fresh AB113 solutions without changing the overall concentration of the catalyst under ultrasonic irradiation. Results provided in Fig. 14 show that the piezo-catalytic activity of the m-S–BaSO_4_–BaTiO_3_ sample does not show a significant loss after five recycles for the degradation of AB113.

## Conclusion

In this research, the piezocatalytic activity of barium sulfate (a very cheap mineral) improved by coupling it with BaTiO_3_ and doped BaTiO_3_ (Cu–BaTiO_3_, Fe–BaTiO_3_, S–BaTiO_3_, and N–BaTiO_3_) in the BaSO_4_–BaTiO_3_ composite. Cu–BaSO_4_–BaTiO_3_, Fe–BaSO_4_–BaTiO_3_, S–BaSO_4_–BaTiO_3_, and N–BaSO_4_–BaTiO_3_ were modified by sucrose as a natural piezo-material to achieve more improvement.

SEM and EDX results show that BaSO_4_ appeared as micro-size particles, while BaTiO_3_ and Cu–BaTiO_3_, Fe–BaTiO_3_, S–BaTiO_3_, and N–BaTiO_3_ appeared as nano-sized structures. Ba source for preparing BaTiO_3_ came from initial BaSO_4_. XRD and EDX do not support doping N into BaTiO_3_, this could be happening because it does not dop into BaTiO_3_ or a very low amount of it is doped into BaTiO_3_. Piezocatalytic activity of BaSO_4_, BaSO_4_–BaTiO_3_, X-BaSO_4_–BaTiO_3_ (X: Cu, Fe, S, and N), and X-BaSO_4_–BaTiO_3_ modified by sucrose were studied by the degradation of AR151 and AB113. Ultrasonic irradiation was used as a mechanical force. Results approve that the piezocatalytic activity of BaSO_4_ sufficiently improved by coupling it with BaTiO_3_, X-BaTiO_3_, and their modification by sucrose. For instance, BaSO_4_ degrade 56.7% of AR151 and 60.9% of AB113 during 90 min ultrasonic vibration. By coupling it with BaTiO_3_, degradation efficiency increased to 61.3% and 64.4%, respectively. Doping BaTiO_3_ with copper improves the degradation efficiency of AR151 to 86.7%. Doping BaTiO_3_ with S improves the decontamination efficiency of AB113 to 89.2% which shows a huge enhancement. Modify BaSO_4_–S–BaTiO_3_ with sucrose lead to achieve a degradation efficiency of 93.3% for AB113. Besides, a possible mechanism was disused by using radical trapping experimental.

## Supplementary Information

Supplementary Information.

## Data Availability

All data generated or analysed during this study are included in this published article (and its Supplementary Information files).

## References

[1] Asadzadeh-Khaneghah S, Habibi-Yangjeh A (2020). g-C3N4/carbon dot-based nanocomposites serve as efficacious photocatalysts for environmental purification and energy generation: A review. J. Clean. Prod..

[2] Huang P, Liang Z, Zhao Z, Cui F (2021). Synthesis of hydrotalcite-like compounds with drinking water treatment residuals for phosphorus recovery from wastewater. J. Clean. Prod..

[3] Kumar R, Raizada P, Verma N, Hosseini-Bandegharaei A, Thakur VK, Nguyen V-H, Singh P (2021). Recent advances on water disinfection using bismuth based modified photocatalysts: Strategies and challenges. J. Clean. Prod..

[4] Le Pivert M, Kerivel O, Zerelli B, Leprince-Wang Y (2021). ZnO nanostructures based innovative photocatalytic road for air purification. J. Clean. Prod..

[5] Tavker N, Sharma M (2020). Fruit rinds extracted cellulose and its utility in fabricating visible light tin sulfide photocatalyst for the treatment of dye, pharmaceutical and textile effluents. J. Clean. Prod..

[6] Wang Y, Sun H, Ang HM, Tadé MO, Wang S (2014). Magnetic Fe3O4/carbon sphere/cobalt composites for catalytic oxidation of phenol solutions with sulfate radicals. Chem. Eng. J..

[7] Kabra K, Chaudhary R, Sawhney R (2007). Effect of pH on solar photocatalytic reduction and deposition of Cu (II), Ni (II), Pb (II) and Zn (II): Speciation modeling and reaction kinetics. J. Hazard. Mater..

[8] Pereira JH, Queirós DB, Reis AC, Nunes OC, Borges MT, Boaventura RA, Vilar VJ (2014). Process enhancement at near neutral pH of a homogeneous photo-Fenton reaction using ferricarboxylate complexes: Application to oxytetracycline degradation. Chem. Eng. J..

[9] Sun J, Li X, Feng J, Tian X (2009). Oxone/Co2+ oxidation as an advanced oxidation process: comparison with traditional Fenton oxidation for treatment of landfill leachate. Water Res..

[10] Kilic MY, Abdelraheem WH, He X, Kestioglu K, Dionysiou DD (2019). Photochemical treatment of tyrosol, a model phenolic compound present in olive mill wastewater, by hydroxyl and sulfate radical-based advanced oxidation processes (AOPs). J. Hazard. Mater..

[11] Mansouri L, Tizaoui C, Geissen S-U, Bousselmi L (2019). A comparative study on ozone, hydrogen peroxide and UV based advanced oxidation processes for efficient removal of diethyl phthalate in water. J. Hazard. Mater..

[12] Sharma VK, Feng M (2019). Water depollution using metal-organic frameworks-catalyzed advanced oxidation processes: a review. J. Hazard. Mater..

[13] Beshkar F, Amiri O, Salehi Z (2017). Synthesis of ZnSnO3 nanostructures by using novel gelling agents and their application in degradation of textile dye. Sep. Purif. Technol..

[14] Ebadi M, Amiri O, Sabet M (2018). Synthesis of CeO2/Au/Ho nanostructures as novel and highly efficient visible light driven photocatalyst. Sep. Purif. Technol..

[15] Kumar C, Gaur A, Tiwari S, Biswas A, Rai SK, Maiti P (2019). Bio-waste polymer hybrid as induced piezoelectric material with high energy harvesting efficiency. Compos. Commun..

[16] Surmenev RA, Orlova T, Chernozem RV, Ivanova AA, Bartasyte A, Mathur S, Surmeneva MA (2019). Hybrid lead-free polymer-based nanocomposites with improved piezoelectric response for biomedical energy-harvesting applications: A review. Nano Energy.

[17] Tu D, Xu CN, Yoshida A, Fujihala M, Hirotsu J, Zheng XG (2017). LiNbO3: Pr3+: a multipiezo material with simultaneous piezoelectricity and sensitive piezoluminescence. Adv. Mater..

[18] Zhong H, Xia J, Wang F, Chen H, Wu H, Lin S (2017). Graphene-piezoelectric material heterostructure for harvesting energy from water flow. Adv. Funct. Mater..

[19] Li P, Zhai J, Shen B, Zhang S, Li X, Zhu F, Zhang X (2018). Ultrahigh piezoelectric properties in textured (K, Na) NbO3-based lead-free ceramics. Adv. Mater..

[20] Zheng T, Wu J, Xiao D, Zhu J (2018). Recent development in lead-free perovskite piezoelectric bulk materials. Prog. Mater Sci..

[21] Ma J, Chen X, Lian H, Zhang Q, Liu J (2019). Microstructure, dielectric, piezoelectric, and ferroelectric properties of fine-grained 0.94 Na_0.5_Bi_0.5_TiO_3_-0.06 BaTiO_3_ ceramics. J. Eur. Ceram. Soc..

[22] Shuai C, Liu G, Yang Y, Yang W, He C, Wang G, Liu Z, Qi F, Peng S (2020). Functionalized BaTiO_3_ enhances piezoelectric effect towards cell response of bone scaffold. Colloids Surf., B.

[23] Seshadri SB, Nolan MM, Tutuncu G, Forrester JS, Sapper E, Esteves G, Granzow T, Thomas PA, Nino JC, Rojac T (2018). Unexpectedly high piezoelectricity of Sm-doped lead zirconate titanate in the Curie point region. Sci. Rep..

[24] Hamid HA, Çelik-Butler Z (2018). Characterization and performance analysis of Li-doped ZnO nanowire as a nano-sensor and nano-energy harvesting element. Nano Energy.

[25] Chen X, Wang L, Jia Y, Li Y, Li X, Dong W, Wang B, Wang X (2019). Strongly enhanced ferroelectric performance in Ca-doped barium titanate coatings produced by plasma electrolytic oxidation. Ceram. Int..

[26] Lee KY, Kumar B, Seo J-S, Kim K-H, Sohn JI, Cha SN, Choi D, Wang ZL, Kim S-W (2012). P-type polymer-hybridized high-performance piezoelectric nanogenerators. Nano Lett..

[27] Pradel KC, Wu W, Zhou Y, Wen X, Ding Y, Wang ZL (2013). Piezotronic effect in solution-grown p-type ZnO nanowires and films. Nano Lett..

[28] Shin S-H, Kim Y-H, Lee MH, Jung J-Y, Seol JH, Nah J (2014). Lithium-doped zinc oxide nanowires–polymer composite for high performance flexible piezoelectric nanogenerator. ACS Nano.

[29] Ul R, Marchet P, Pham-Thi M, Tran-Huu-Hue L-P (2019). Improved properties of doped BaTiO_3_ piezoelectric ceramics. Phys. Status Solidi (a).

[30] Dhananjay D, Nagaraju J, Krupanidhi S (2007). Off-centered polarization and ferroelectric phase transition in Li-doped ZnO thin films grown by pulsed-laser ablation. J. Appl. Phys..

[31] Yang Y, Song C, Wang X, Zeng F, Pan F (2008). Giant piezoelectric d 33 coefficient in ferroelectric vanadium doped ZnO films. Appl. Phys. Lett..

[32] Yin J, Zhang G, Liu H, Liang J (2013). Hydrothermal fabrication and ferroelectric behavior of lithium-doped zinc oxide nanoflakes. Sci. Adv. Mater..

[33] Liu G, Abdel-Rahman E, Ban D (2015). Performance optimization of pn homojunction nanowire-based piezoelectric nanogenerators through control of doping concentration. J. Appl. Phys..

[34] Kaur J, Shah J, Kotnala RK, Verma KCh (2012). Raman spectra, photoluminescence and ferromagnetism of pure, Co and Fe doped SnO2 nanoparticles. Ceram. Int..

[35] Chen KJ, Fang TH, Hung FY, Ji LW, Chang SJ, Young SJ, Hsiao YJ (2008). The crystallization and physical properties of Al-doped ZnO nanoparticles. Appl. Surf. Sci..

[36] Kumari R, Sahai A, Goswami N (2015). Effect of nitrogen doping on structural and optical properties of ZnO nanoparticles. Prog. Nat. Sci. Mater. Int..

[37] Nandakumar N, Kurian P (2012). Chemosynthesis of monodispersed porous BaSO4 nano powder by polymeric template process and its characterisation. Powder Technol..

[38] Lee J, Cha S, Kim J, Nam H, Lee S, Ko W, Wang KL, Park J, Hong J (2011). p-Type conduction characteristics of lithium-doped ZnO nanowires. Adv. Mater..

[39] Kim S, Rahman T, Senesac LR, Davison BH, Thundat T (2009). Piezoresistive cantilever array sensor for consolidated bioprocess monitoring. Scanning.

[40] Jiao G, Fan H, Liu L, Wang W (2007). Structure and piezoelectric properties of Cu-doped potassium sodium tantalate niobate ceramics. Mater. Lett..

[41] Ong WL, Huang H, Xiao J, Zeng K, Ho GW (2014). Tuning of multifunctional Cu-doped ZnO films and nanowires for enhanced piezo/ferroelectric-like and gas/photoresponse properties. Nanoscale.

[42] Watson BH, Brova MJ, Fanton MA, Meyer RJ, Messing GL (2020). Mn-and Mn/Cu-doped PIN-PMN-PT piezoelectric ceramics for high-power transducers. J. Am. Ceram. Soc..

[43] Babakr, A. B., Amiri, O., Guo, L. J., Rashi, M. A. & Mahmood, P. H. Kinetic and thermodynamic study in piezo degradation of methylene blue by SbSI/Sb2S3 nanocomposites stimulated by zirconium oxide balls. *Sci. Rep.* **12**, Article number: 15242 (2022). 10.1038/s41598-022-19552-3 PMC9463189 36085338

[44] Amiri O, Beshkar F, Ahmed SS, Rafiei-Miandashti A, Mahmood PH, Dezaye AA (2021). Novel flower-like (Bi (Bi2S3) 9I3) 2/3 nanostructure as efficient photocatalyst for photocatalytic desulfurization of benzothiophene under visible light irradiation. Adv. Powder Technol..

[45] Amiri O, Salar K, Othman P, Rasul T, Faiq D, Saadat M (2020). Purification of wastewater by the piezo-catalyst effect of PbTiO_3_ nanostructures under ultrasonic vibration. J. Hazard. Mater..

[46] Bai Y, Jian J, Liu D, Zhao X (2021). Synthesis, characterization and application of a new biomass-based antioxidant derived from vanillin and methyl ethyl ketone. J. Clean. Prod..

[47] Qian H, Hou Q, Yu G, Nie Y, Bai C, Bai X, Ju M (2020). Enhanced removal of dye from wastewater by Fenton process activated by core-shell NiCo2O4@ FePc catalyst. J. Clean. Prod..

