# Supplementary material for: Boost piezocatalytic activity of BaSO4 by coupling it with BaTiO3, Cu:BaTiO3, Fe:BaTiO3, S:BaTiO3 and modify them by sucrose for water purification
Source: Sci Rep. 2022 Dec 1;12:20792. doi: 10.1038/s41598-022-24992-y (PMC9715647; doi:10.1038/s41598-022-24992-y)
Supplement: Supplementary file 1 — Supplementary Information. [file 41598_2022_24992_MOESM1_ESM.pdf]

Boost Piezocatalytic activity of $BaSO_4$ by coupling it with $BaTiO_3$, Cu:$BaTiO_3$, Fe:$BaTiO_3$, S:$BaTiO_3$ and modify them by sucrose for water purification

Fe:$BaTiO_3$, S:$BaTiO_3$ and modify them by sucrose for water purification

Omid Amiri [a, b, *], Gashaw L. Abdulla [b], Chnar M. Burhan [b], Hawnaz H. Hussein [b], Amir Mahyar Azhdarpour [c], Mohsen Saadat [d], Mohammad Joshaghani [a, **], Peshawa H. Mahmood [b]

[a] Faculty of Chemistry, Razi University, Kermanshah 67149, Iran

[b] Chemistry Department, College of Science, University of Raparin, Rania, Kurdistan Region, Iraq

[c] Applied Geological Research Center of Iran, Karaj Iran

[d] Department of Physics, University of Sistan and Baluchestan, Zahedan, Iran

* Corresponding author. Tel: +9647700581175

E-mail address: o.amiri1@gmail.com, oamiri@uor.edu.krd

** Corresponding author E-mail Address: mjoshaghani@razi.ac.ir

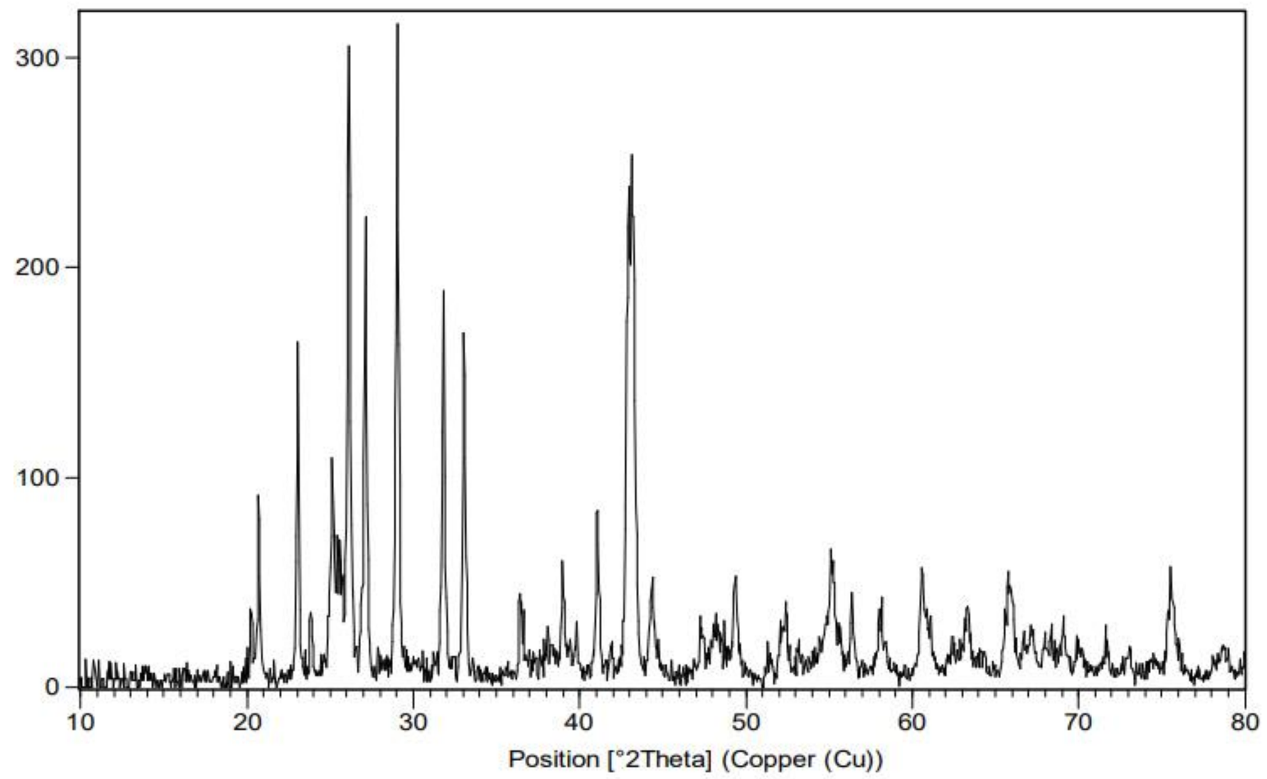

Fig. S1. XRD pattern of $BaSO_4$-$BaTiO_3$.

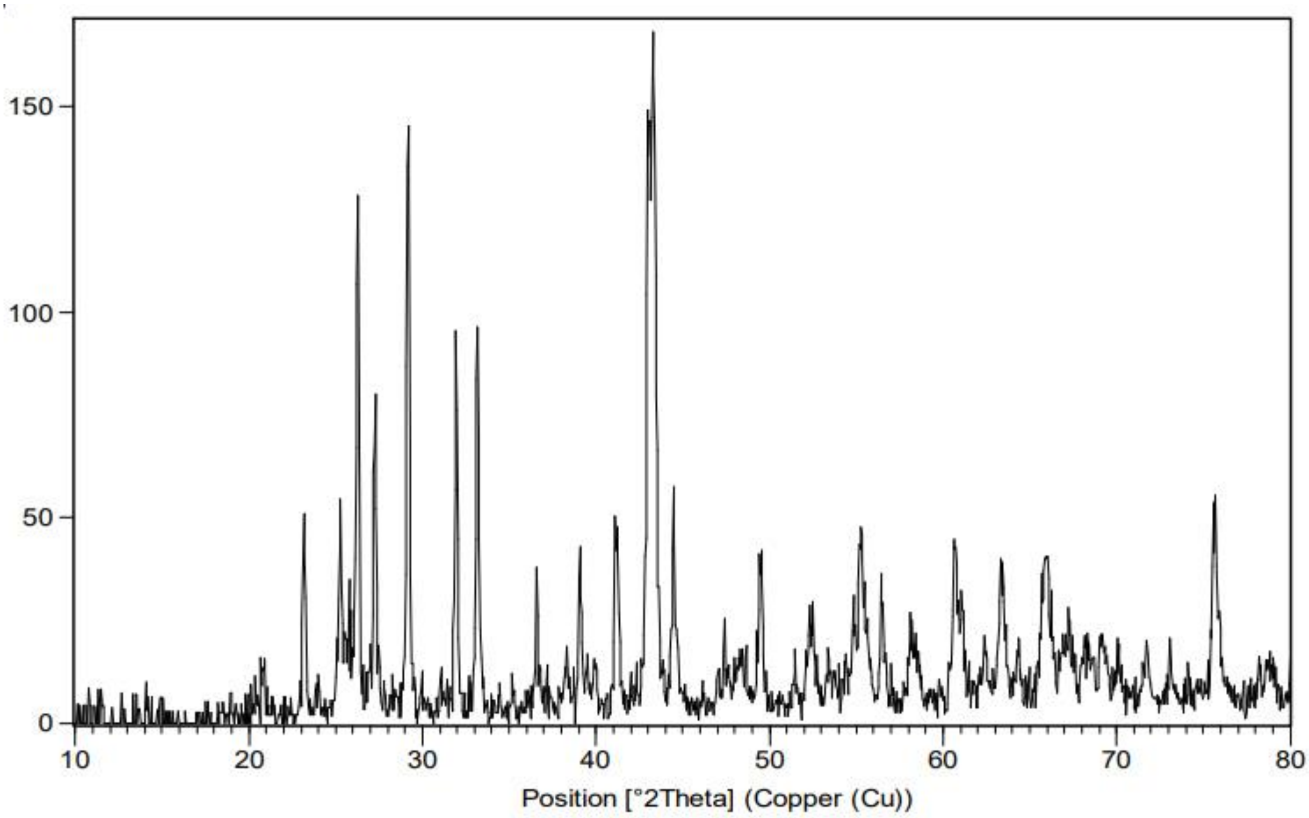

Fig. S2. XRD pattern of Cu-$BaSO_4$-$BaTiO_3$.

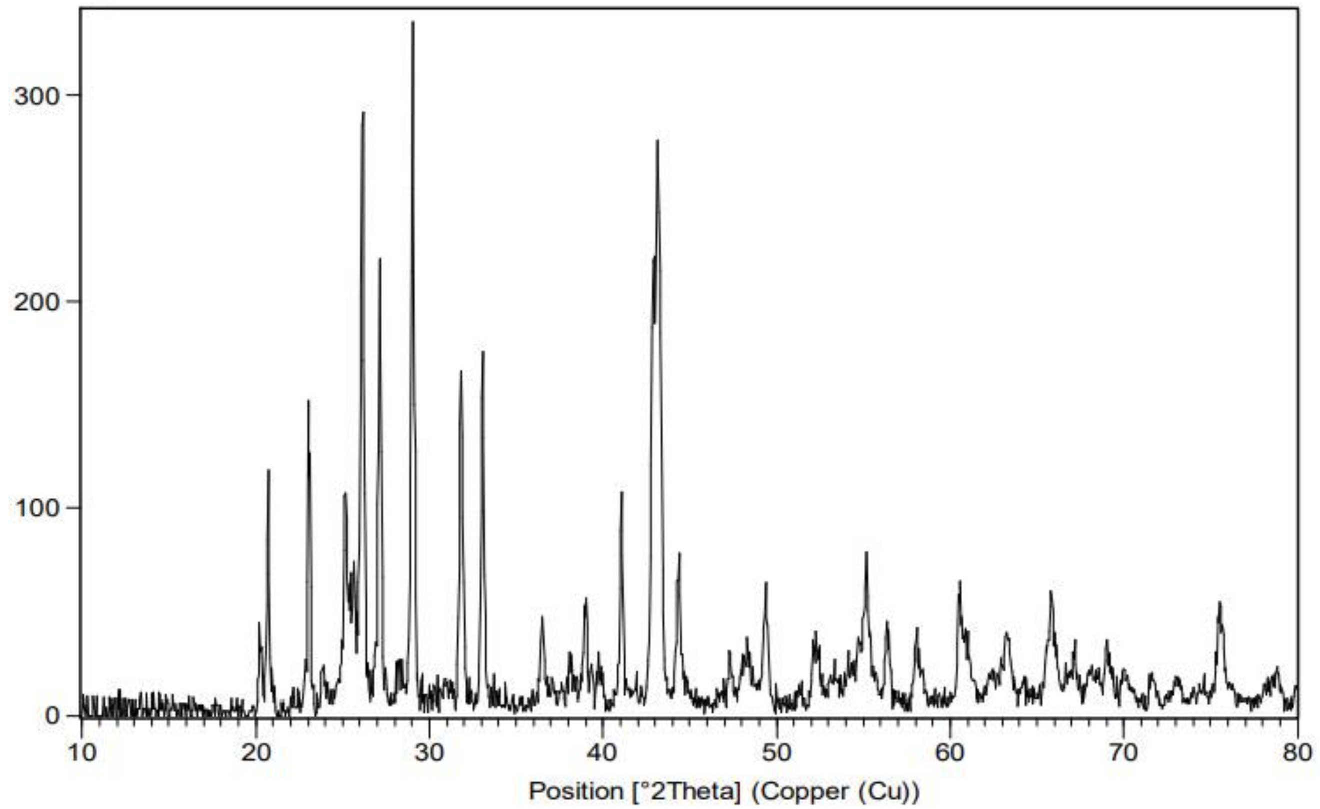

Fig. S3. XRD pattern of Fe-$BaSO_4$-$BaTiO_3$.

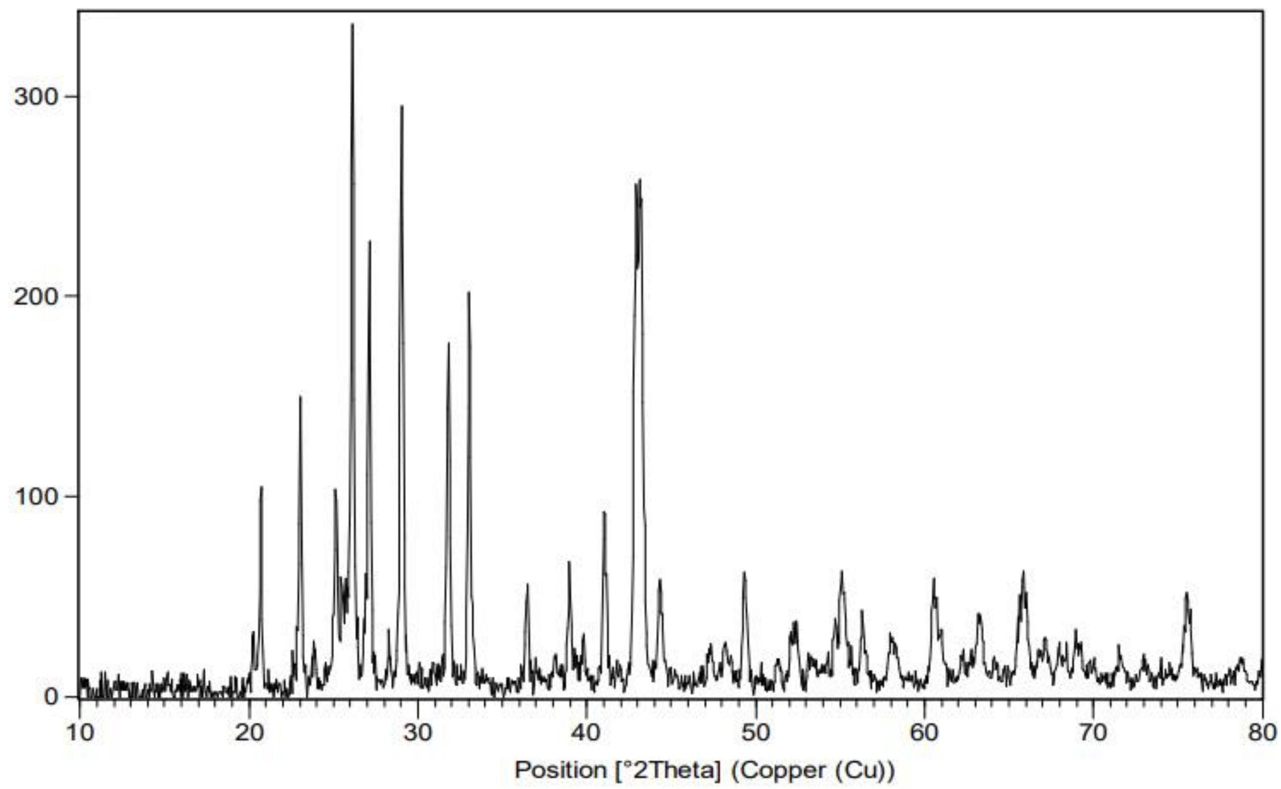

Fig. S4. XRD pattern of S-$BaSO_4$-$BaTiO_3$.

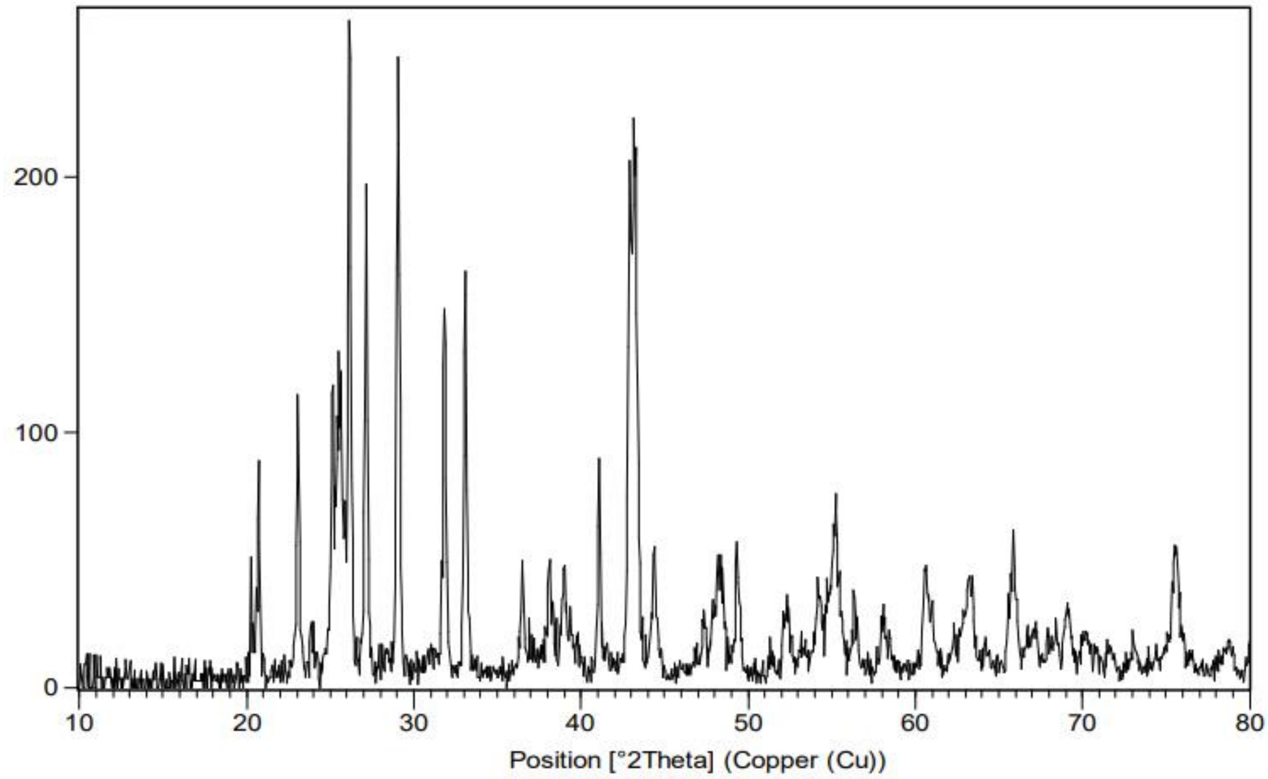

Fig. S5. XRD pattern of N-$BaSO_4$-$BaTiO_3$.

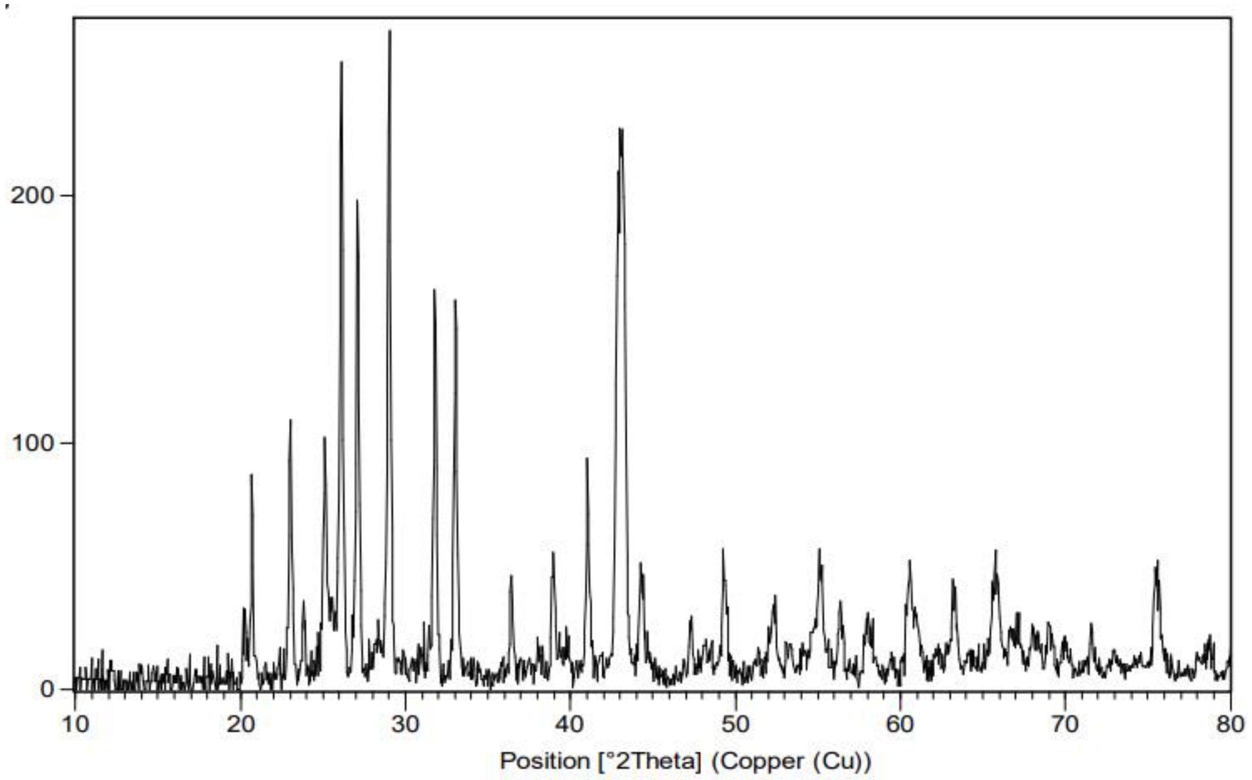

Fig. S6. XRD pattern of m-S-$BaSO_4$-$BaTiO_3$.

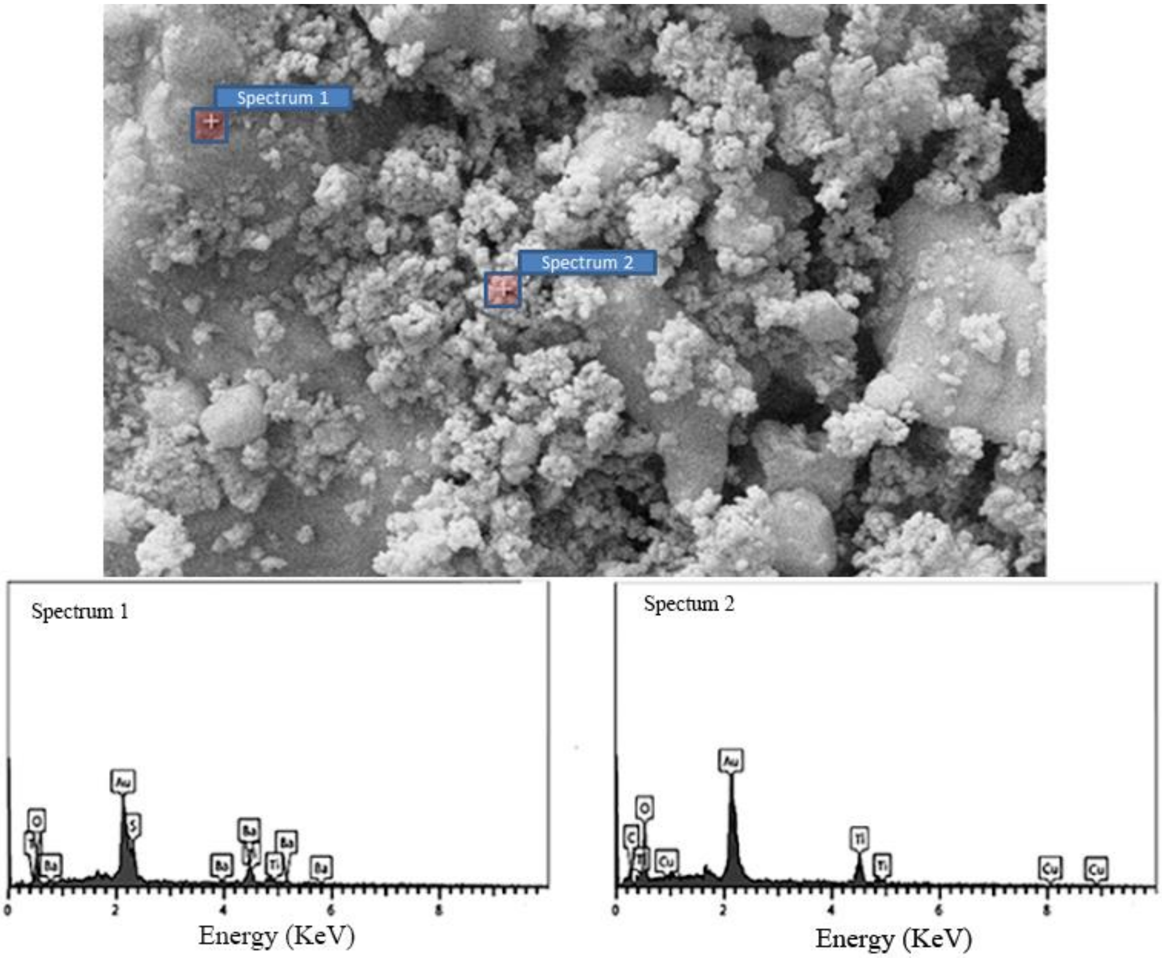

Fig. S7. SEM image and related EDX spectra of Cu-$BaSO_4$-$BaTiO_3$. Spectrum 1 show the element for micro size particle and spectrum 2 show the elements for Nano size particles.

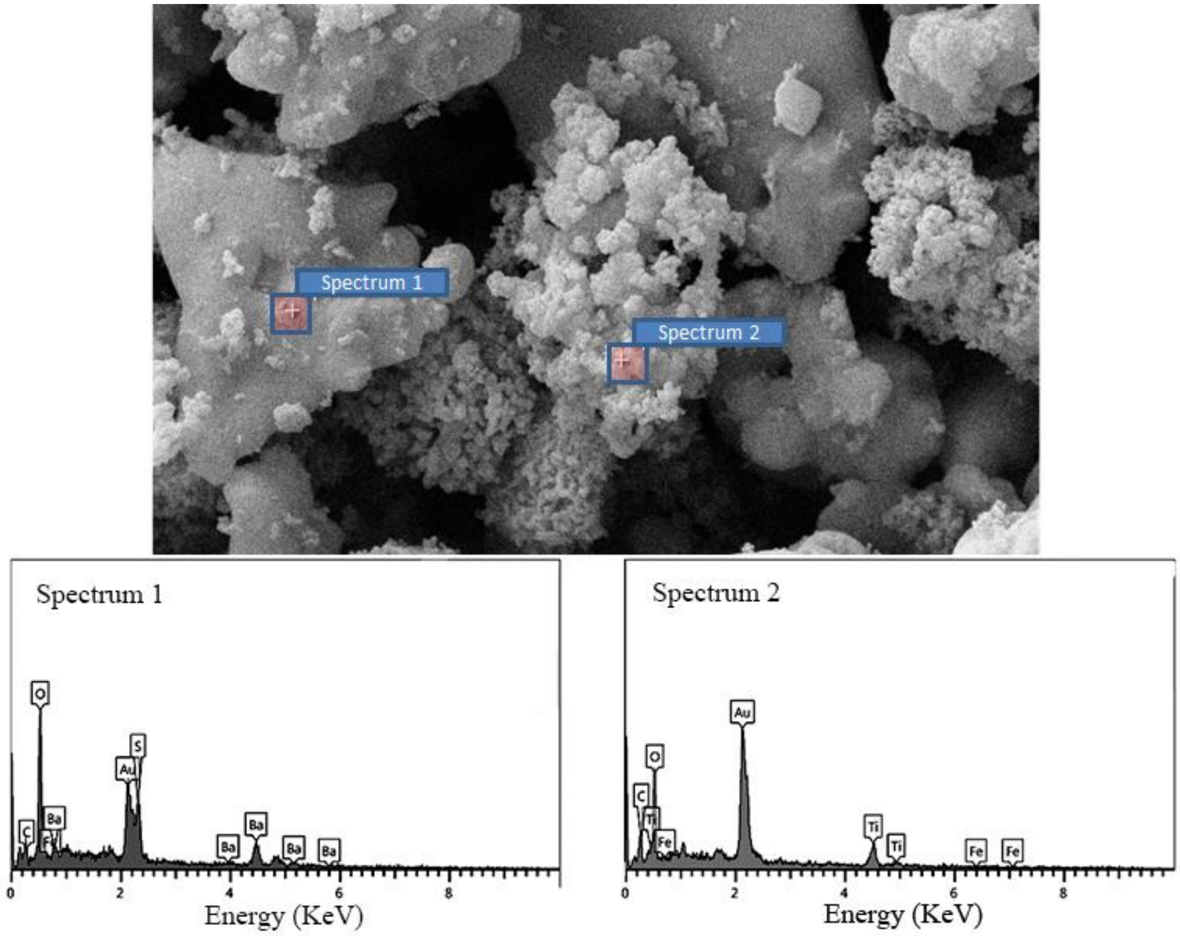

Fig. S8. SEM image and related EDX spectra of Fe-$BaSO_4$-$BaTiO_3$. Spectrum 1 show the element for micro size particle and spectrum 2 show the elements for Nano size particles.

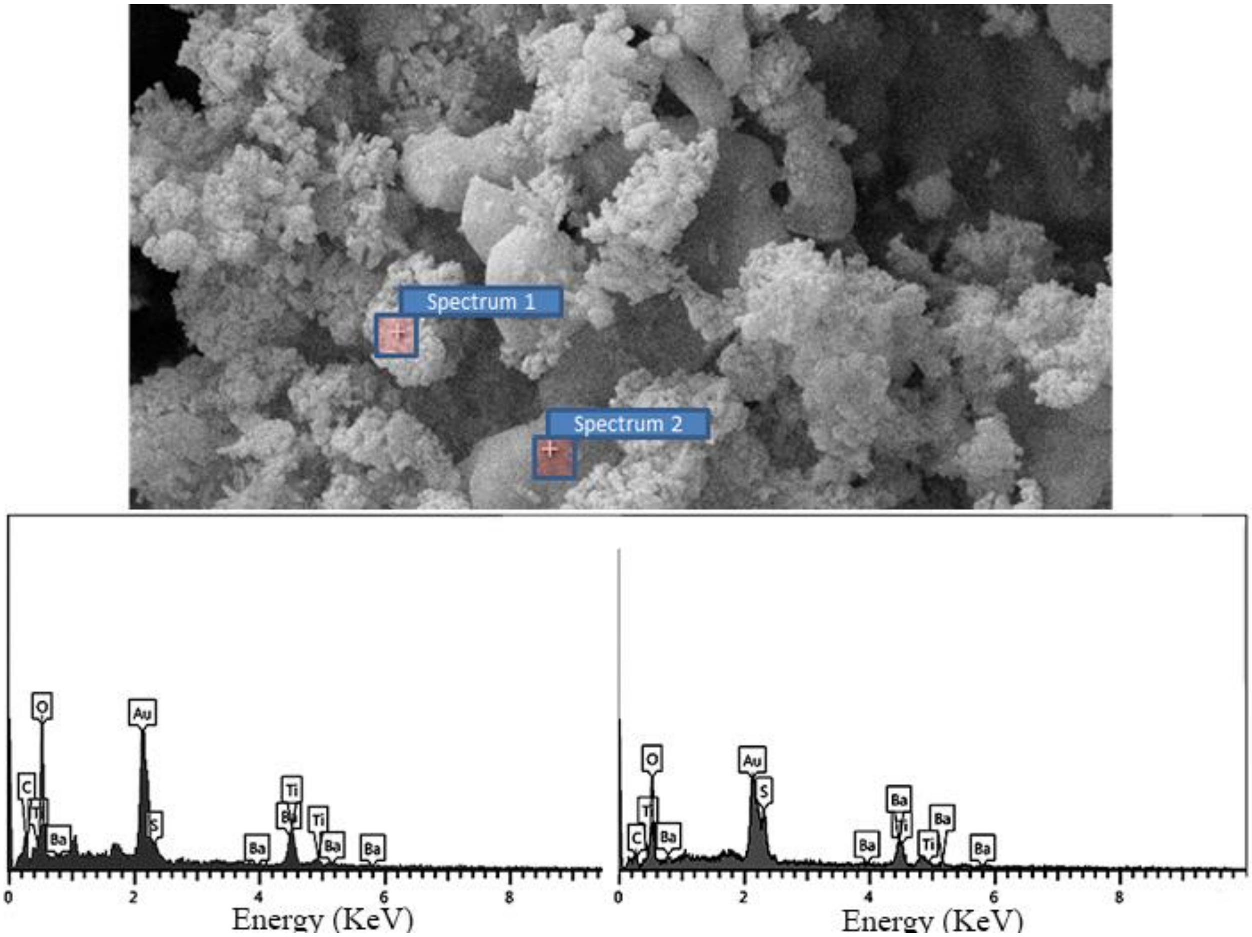

Fig. S9. SEM image and related EDX spectra of S-$BaSO_4$-$BaTiO_3$. Spectrum 1 show the element for micro size particle and spectrum 2 show the elements for Nano size particles.

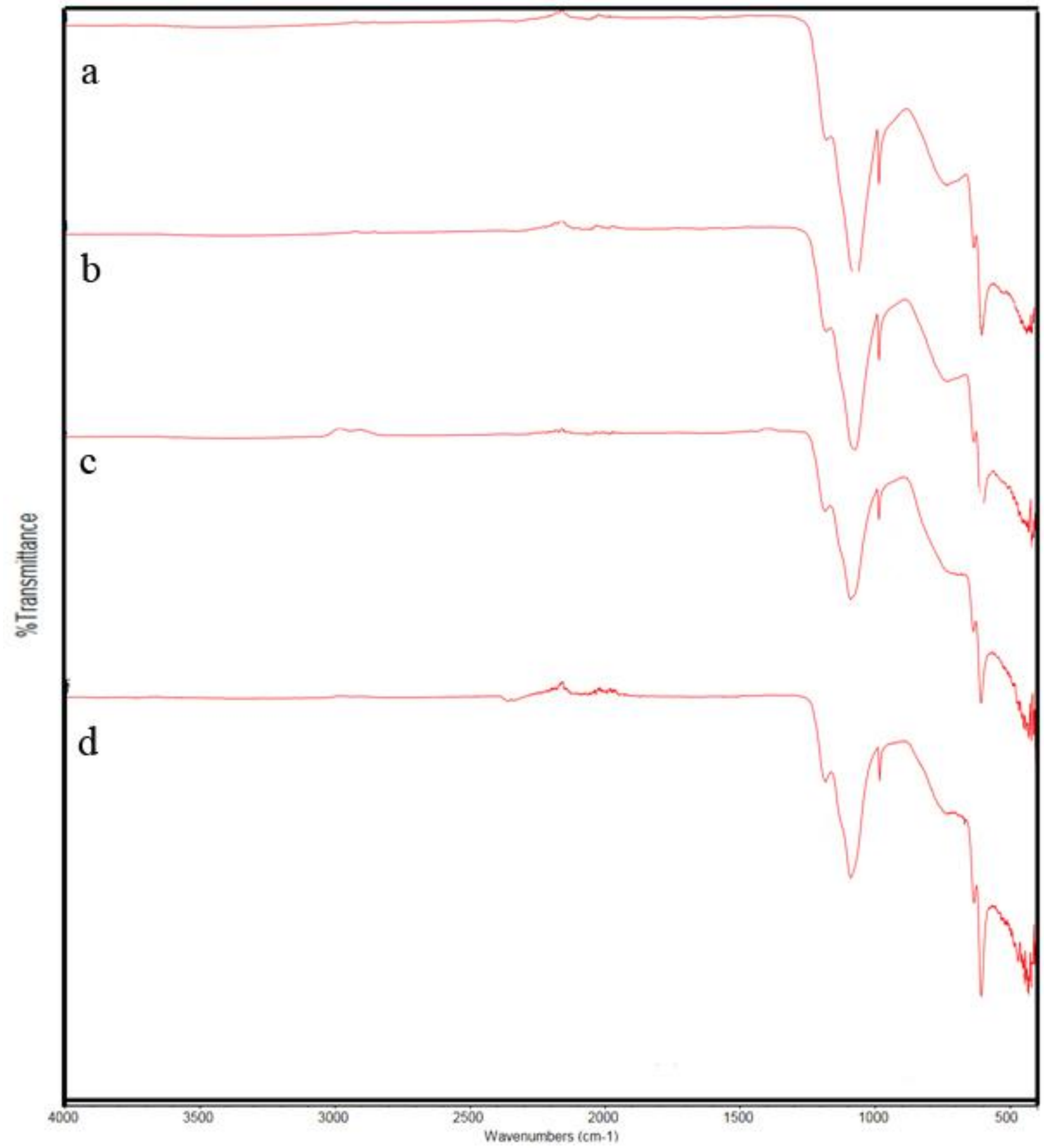

Fig. S10. FT-IR of Cu-$BaSO_4$-$BaTiO_3$, Fe-$BaSO_4$-$BaTiO_3$, N-$BaSO_4$-$BaTiO_3$ and S-$BaSO_4$-$BaTiO_3$.

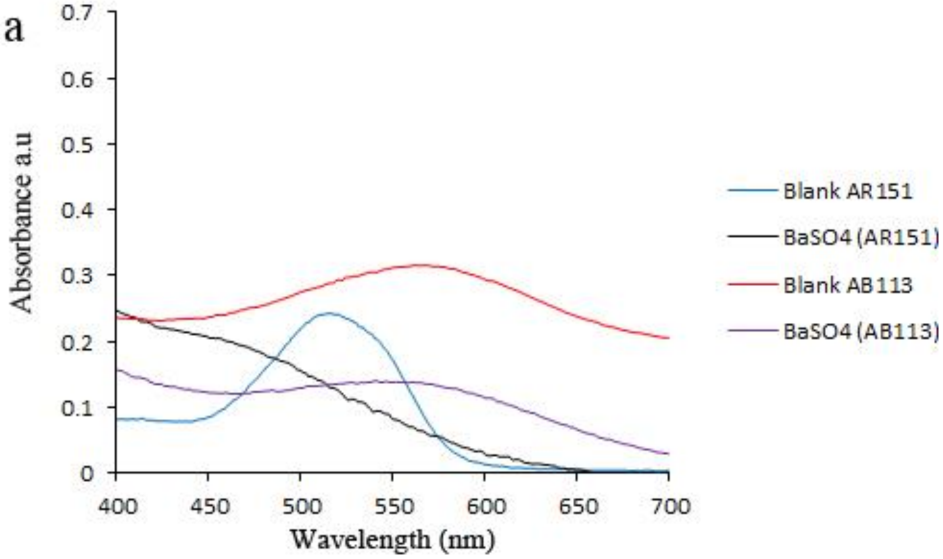

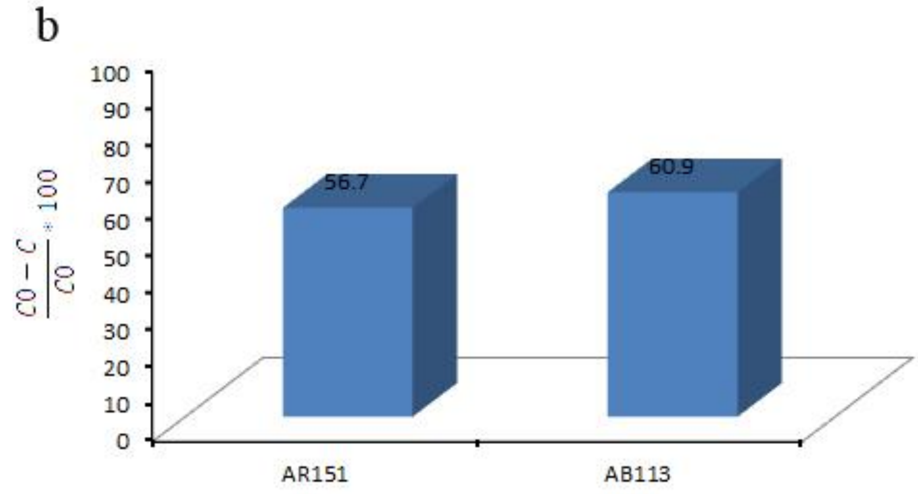

Fig. S 11. **a)** The UV-Vis spectrum of initial AR151, initial AB113, AR151 after treat it with Pure $BaSO_4$, and AB113 after treat it with Pure $BaSO_4$. b) Degradation efficiency by using Pure $BaSO_4$ for AR151 and AB113.
